# Kinetic analysis of paramyxovirus-sialoglycan receptor interactions reveals virion motility

**DOI:** 10.1371/journal.ppat.1011273

**Published:** 2023-03-27

**Authors:** Xuesheng Wu, Maite Goebbels, Lemeng Chao, Tom Wennekes, Frank J. M. van Kuppeveld, Erik de Vries, Cornelis A. M. de Haan

**Affiliations:** 1 Section Virology, Division Infectious Diseases and Immunology, Department Biomolecular Health Sciences, Faculty Veterinary Medicine, Utrecht University, Utrecht, the Netherlands; 2 Department Chemical Biology and Drug Discovery, Utrecht Institute for Pharmaceutical Sciences and Bijvoet Center for Biomolecular Research, Utrecht University, Utrecht, The Netherlands; Brown University, UNITED STATES

## Abstract

Many viruses initiate infection by binding to sialoglycan receptors at the cell surface. Binding to such receptors comes at a cost, however, as the sheer abundance of sialoglycans e.g. in mucus, may immobilize virions to non-functional decoy receptors. As a solution, sialoglycan-binding as well as sialoglycan-cleavage activities are often present in these viruses, which for paramyxoviruses are combined in the hemagglutinin-neuraminidase (HN) protein. The dynamic interactions of sialoglycan-binding paramyxoviruses with their receptors are thought to be key determinants of species tropism, replication and pathogenesis. Here we used biolayer interferometry to perform kinetic analyses of receptor interactions of animal and human paramyxoviruses (Newcastle disease virus, Sendai virus, and human parainfluenza virus 3). We show that these viruses display strikingly different receptor interaction dynamics, which correlated with their receptor-binding and -cleavage activities and the presence of a second sialic acid binding site. Virion binding was followed by sialidase-driven release, during which virions cleaved sialoglycans until a virus-specific density was reached, which was largely independent of virion concentration. Sialidase-driven virion release was furthermore shown to be a cooperative process and to be affected by pH. We propose that paramyxoviruses display sialidase-driven virion motility on a receptor-coated surface, until a threshold receptor density is reached at which virions start to dissociate. Similar motility has previously been observed for influenza viruses and is likely to also apply to sialoglycan-interacting embecoviruses. Analysis of the balance between receptor-binding and -cleavage increases our understanding of host species tropism determinants and zoonotic potential of viruses.

## Author summary

Many viruses attach to host cells using glycan receptors. These receptors are, however, extremely abundant in mucus, while also not all glycans on the cell surface will act as functional receptors. To prevent getting stuck to decoy receptors such viruses often contain in addition to a receptor-binding protein also a receptor-destroying enzyme. Several paramyxoviruses contain a hemagglutinin-neuraminidase, which combines sialoglycan receptor-binding and -cleavage properties in a single catalytic site. Here, we applied biolayer interferometry (BLI) to study the kinetics of the interactions between several human and animal paramyxoviruses and sialoglycan receptors. With BLI we can monitor in real time the binding and release of viruses with glycan receptors coated on a sensor surface. We show that the dynamics of these interactions may differ dramatically between viruses when using the same receptors. Dynamic interactions allow virus particle motility on a receptor-coated surface driven by sialidase activity and thus escape from decoy receptors. We propose that different virus-receptor dynamics evolve upon adaptation to different host species and that the balance between receptor binding and cleavage is an important determinant of host species tropism.

## Introduction

Virus infection starts with interaction of virions with cell surface receptors. Sialoglycans are receptors for many viruses, including influenza A-D viruses, several coronaviruses and paramyxoviruses [1]. Sialoglycan abundance on non-functional/decoy receptors, for example in mucus, poses a risk for viruses as they may become immobilized before reaching functional receptors on host cells. Therefore, the above-mentioned viruses also carry receptor-destroying activity. Evasion of decoy receptors and binding to functional receptors at the cell surface is a key determinant of host tropism and viral pathogenesis [2–7] and requires a better understanding of the dynamics of virus-sialoglycan receptor interactions. While recent advances have been made concerning the dynamics of influenza virus-receptor interactions [6–12], much less is known about these processes for sialoglycan-binding paramyxoviruses.

The *Paramyxoviridae* family harbors numerous human and animal viruses that are transmitted often via airborne routes [13] and cause a great burden on human and animal health and global economics. Paramyxoviruses are enveloped, negative-stand RNA viruses containing two major envelope proteins: a fusion protein (F) and a receptor-binding protein (RBP). The RBPs either constitute an hemagglutinin-neuraminidase (HN), hemagglutinin (H) or glycoprotein (G). H and G bind to proteinaceous receptors, while HN interacts with sialoglycan receptors. The *Paramyxoviridae* family is divided into four subfamilies and 16 genera according to International Committee on Virus Taxonomy [14]. Viruses belonging to the *Avulavirinae*, genus *Orthorubulavirus* of the *Rubulavirinae*, and genus *Respirovirus* of the *Orthoparamyxovirinae* possess HN glycoproteins. These include among others human respirovirus 3 (better known as human parainfluenza virus 3 [hPIV3]), and animal pathogens such as Sendai virus (SeV; murine respirovirus 1) and Newcastle disease virus (NDV; avian orthoavulavirus 1).

HN is a type II integral membrane protein and is reported to form tetramers or dimers [15,16]. It has a short N-terminal cytoplasmic tail, transmembrane domain, stalk region and C-terminal six-bladed β-propeller head domain typical of sialidases, which contains the catalytic site. The HN catalytic site functions both in sialoglycan receptor binding and cleavage [17–19]. The stalk domain of HN physically interacts with the F protein and plays a role in triggering the F protein rearrangements leading to membrane fusion [15]. Some paramyxoviruses, including NDV contain a 2^nd^ sialic acid (Sia) receptor binding site (2SBS) located at the dimer interface [20,21]. The presence of a 2SBS in SeV HN remains inconclusive. Some results suggests that binding and cleavage activity of SeV HN involves two distinct sites, but in this case structural evidence for the presence of a 2SBS is lacking [22]. hPIV3 appears to lack a 2SBS as a single site was responsible for both binding and cleavage functions [20,23]. In general, HN-containing paramyxoviruses, including those infecting humans, appear to prefer binding to receptors with terminal NeuAc*α*2-3Gal linkage (α2-3-linked Sia) [24–26], in contrast to human influenza A viruses that prefer to bind glycan receptors with terminal NeuAcα2-6Gal linkages (α2-6-linked Sia) [27]. Whether the catalytic site and 2SBS of NDV HN differ in their receptor preference is not known.

Besides sialoglycan receptor preference, the balance between receptor-binding and -cleavage is important for influenza A virus (IAV) and embecovirus cell and species tropism [7,28,29]. Presumably such a balance is also of importance for HN-containing paramyxoviruses. Recently, we developed novel assays based on biolayer interferometry (BLI) to study the hemagglutinin (HA)- neuraminidase (NA)-receptor balance of IAVs [6–8]. We showed that multivalent low-affinity HA-receptor interactions in combination with virion-associated sialidase activity of NA result in virion motility. Virion motility and subsequent release from a receptor-coated surface depends on HA-receptor binding strength, the activity of NA and the identity and density of the receptors. IAVs differ from paramyxoviruses in their sialoglycan receptor interactions. Thus, while IAVs carry different envelope glycoproteins for binding and cleavage, for paramyxoviruses a single HN protein, often even a single catalytic site, conducts both functions. In addition the reported affinities of HN proteins for sialoglycan receptors are higher than those of IAV HA (10–500 μM vs 0.3–3 mM) [30–35]. In the current study we applied BLI to gain more insights into the dynamics of paramyxovirus-receptor interactions by performing kinetic analyses using prototype hPIV3, NDV and SeV.

## Results

### Paramyxovirus hemagglutination and sialidase activity

**Fig 1:**
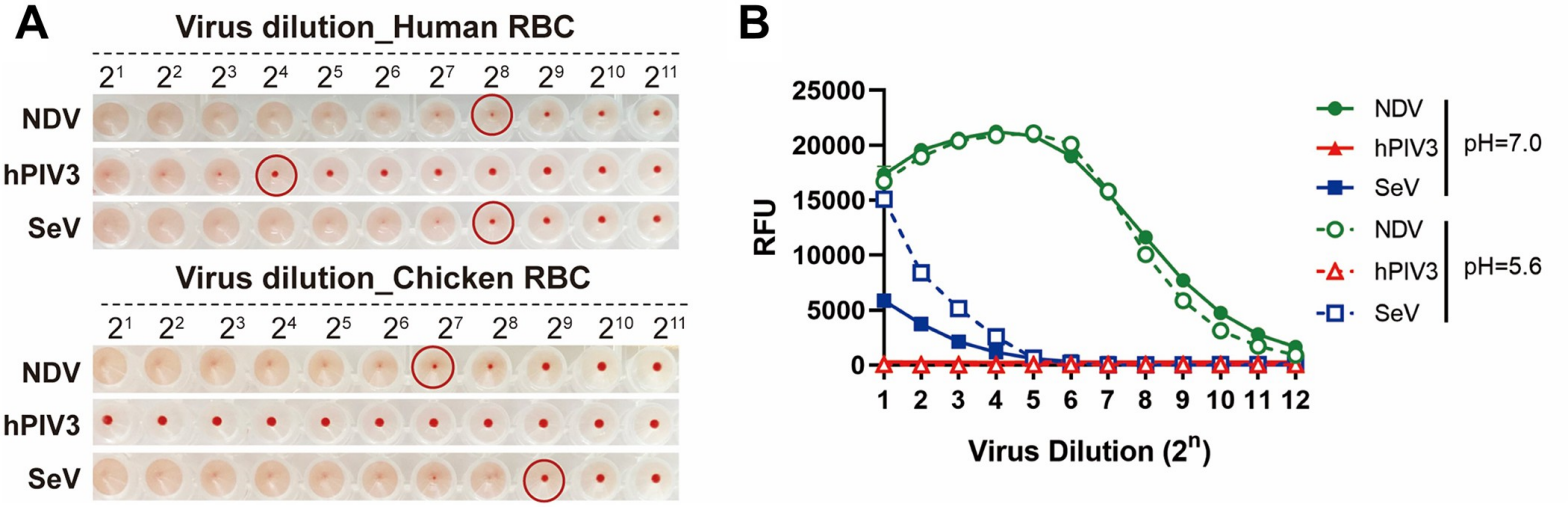
Characterization of virus-receptor interactions using classical techniques. (**A)** Hemagglutination assay. Serial two-fold dilutions of NDV, hPIV3 and SeV (starting concentration 2x10^10^ virus particles/ml as determined by nanoparticle tracking analysis [NTA]) were incubated with human or chicken erythrocytes at 4°C for 2h. A red erythrocyte pellet indicates the absence of hemagglutination. Red circles indicate the first dilution at which no hemagglutination was observed. (**B)** Sialidase activity assay. Sialidase activity of serial two-fold dilutions of NDV, hPIV3 and SeV (starting concentration 2x10^10^ virus particles/ml) was assessed in triplicate at 37°C using the fluorogenic substrate MUNANA at pH 5.6 or 7.0. Means and standard deviations are graphed. The fluorescence generated from MUNANA cleavage was measured using a plate reader (in relative fluorescence units [RFU]).

Prior to analyzing the dynamics of paramyxovirus-receptor interactions using BLI, we examined the receptor interactions of the different viruses in more conventional assays. The ability to bind to sialoglycan receptors was analyzed by a classic hemagglutination assay using both human and chicken red blood cells (RBCs) at 4°C (Fig 1A). Human and chicken erythrocytes express both α2-3- and α2-6-linked Sia, with chicken erythrocytes displaying more α2-3-linked Sia than human RBCs [36]. NDV and SeV agglutinated both human and chicken erythrocytes, while hemagglutinating activity of hPIV3 was weak and only detectable for human RBCs. The sialidase activity of NDV, hPIV3 and SeV was studied using 4-methylumbelliferyl-N-acetylneuraminic acid (MUNANA) as substrate at pH 5.6 or 7.0 (Fig 1B). The sialidase activity of NDV, which was not appreciably affected by pH, was approximately 100–250 fold higher in comparison with SeV. Sialidase activity of SeV was approximately 3-fold higher at pH 5.5 than pH 7.0. Sialidase activity of hPIV3 was too weak to be detected at both pHs using this assay.

### Analysis of receptor interactions by NDV, hPIV3 and SeV using BLI

**Fig 2:**
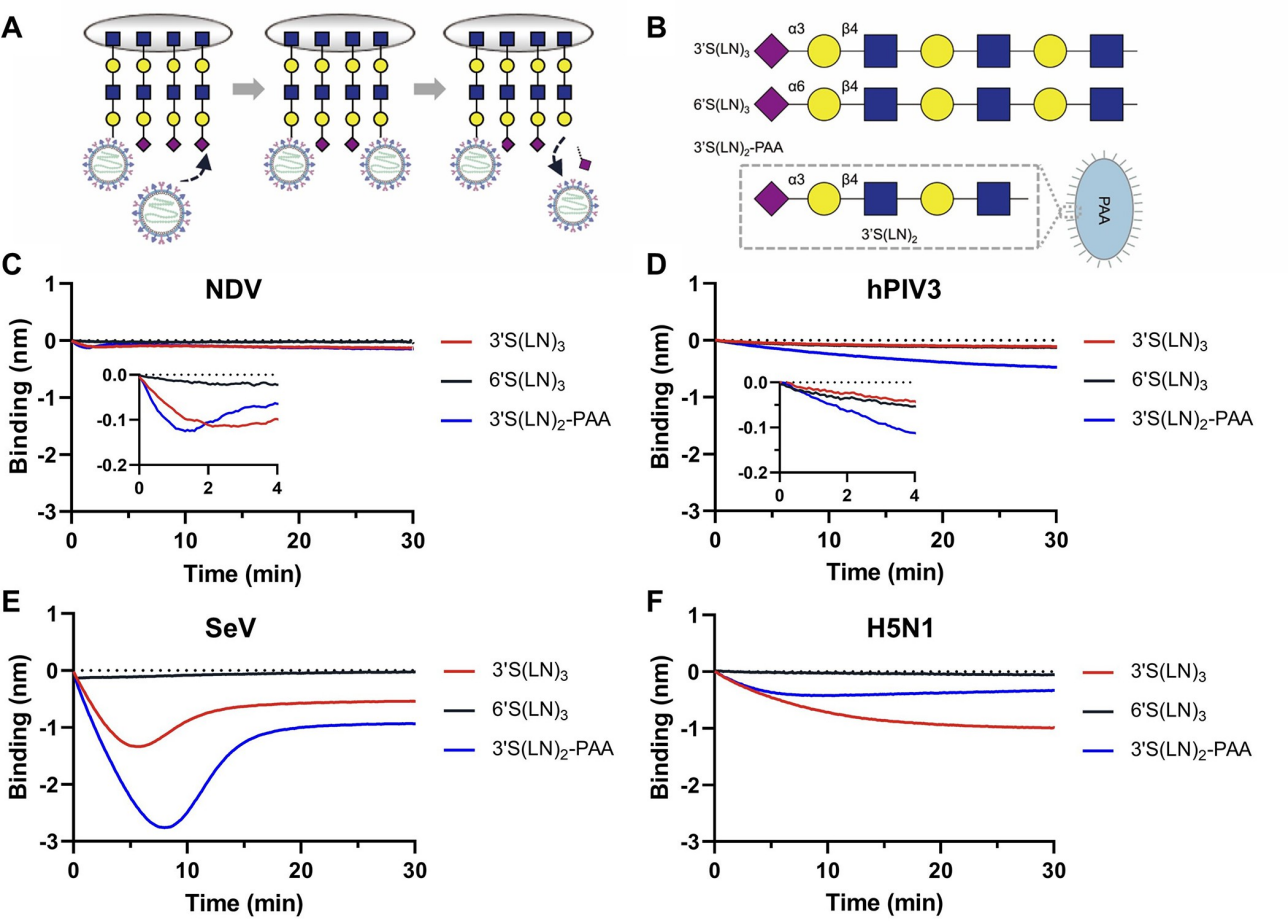
Binding of NDV, hPIV3 and SeV to different sialoglycans. **(A)** Schematic representation of the interaction between virus particles and synthetic biotinylated glycans. **(B)** Biotinylated sialoglycans used in this study are: 3’S(LN)_3_: Neu5Acα2-3Galβ1-4GlcNAcβ1-3Galβ1-4GlcNAcβ1-3Galβ1-4GlcNAc, 6’S(LN)_3_: NeuAcα2-6Galβ1-4GlcNAcβ1-3Galβ1-4GlcNAcβ1-3Galβ1-4GlcNAc; and 3’S(LN)_2_-PAA: Neu5Acα2-3Galβ1-4GlcNAcβ1-3Galβ1-4GlcNAc on a polyacrylamide backbone. Binding curves were generated for NDV (**C**), hPIV3 (**D**), SeV (**E**) and H5N1 (**F**) at 10^10^ particles/ml.

As a first step to analyze the dynamics of paramyxovirus-receptor binding by BLI (Fig 2A), we studied the interaction of NDV, SeV and hPIV3 with different biotinylated synthetic glycans, including 3’S(LN)_3_ (Neu5Acα2-3Galβ1-4GlcNAcβ1-3Galβ1-4GlcNAcβ1-3Galβ1-4GlcNAc), 6’S(LN)_3_ (NeuAcα2-6Galβ1-4GlcNAcβ1-3Galβ1-4GlcNAcβ1-3Galβ1-4GlcNAc), and 3’S(LN)_2_-PAA (Neu5Acα2-3Galβ1-4GlcNAcβ1-3Galβ1-4GlcNAc on a polyacrylamide backbone) (Fig 2B). Sensors loaded with biotin were taken along as controls and used to subtract background signals. Please note that binding of enveloped virions results in a negative binding curve [6] due to the large size of virions similar as observed previously for other vesicles [37]. NDV displayed low binding signals to both 3’S(LN)_3_ and 3’S(LN)_2_-PAA, while no appreciable binding was observed to 6’S(LN)_3_ (Fig 2C). Already after a few (1–3) minutes, binding curves bended, indicative of virion dissociation exceeding association after those time points. hPIV3 and SeV both displayed the highest binding signals to 3’S(LN)_2_-PAA compared to the other glycans (Fig 2D and 2E), with SeV displaying a much higher binding peak than the other viruses. While maximal binding was reached for SeV around 8 min, hPIV3 did not appear to have reached that point after 30 min. Binding of hPIV3 to 3’S(LN)_3_ and 6’S(LN)3 was at a considerable lower, but similar level, while SeV clearly preferred binding to 3’S(LN)_3_ over 6’S(LN)_3_. Binding of virions to 3’S(LN)_2_-PAA treated with bacterial neuraminidase was negligible (S1 Fig). For comparison, the binding of H5N1 avian IAV was analyzed to the same receptors. IAV binding was followed by virus release similar as observed for the paramyxoviruses albeit with different kinetics. No binding was observed to 6’S(LN)_3_. In conclusion, the three paramyxoviruses differ in their virus-receptor interaction kinetics for the receptors studied, with all viruses displaying clear binding signals to 3’S(LN)_2_-PAA receptors.

### Modification of the receptor-coated surface by virion-associated sialidase activity

**Fig 3:**
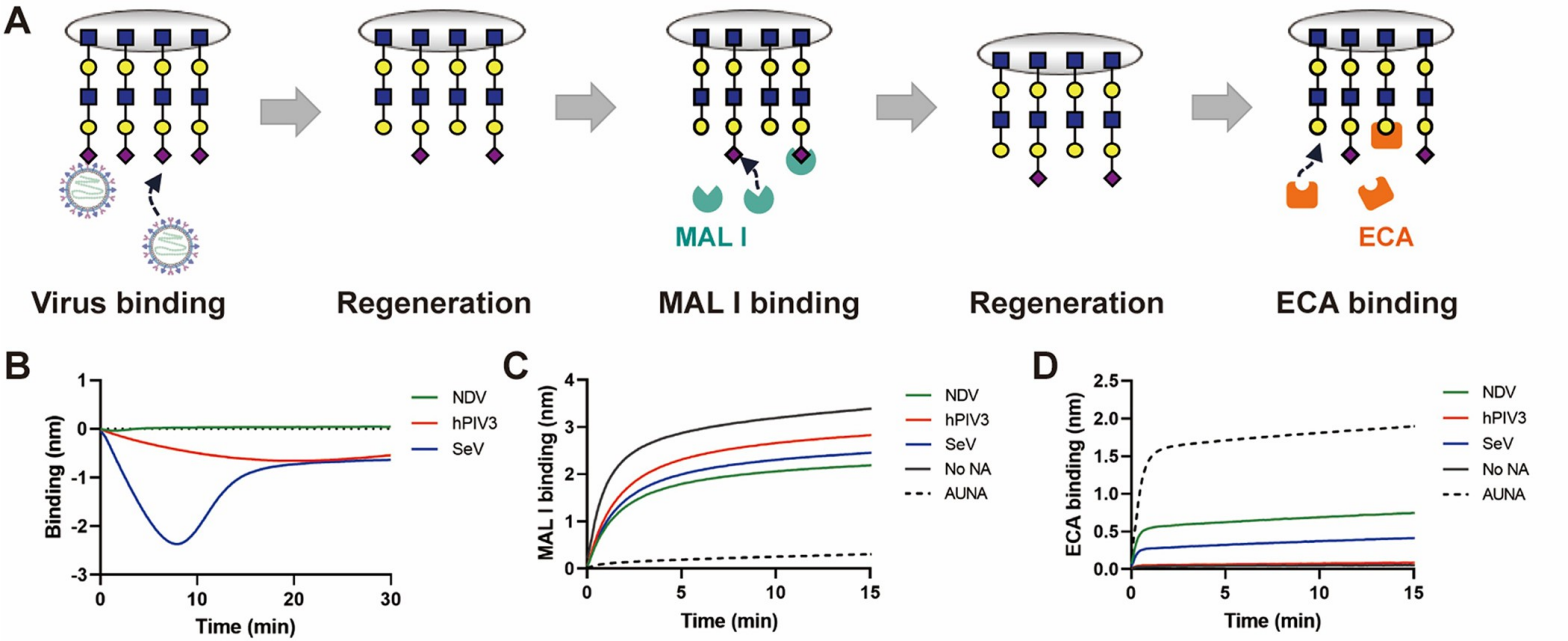
Analysis of NDV, hPIV3, and SeV binding and sialidase activity using lectin binding. (**A)** Schematic representation of virus binding followed by the lectin binding assay. MAL I specifically binds to the remaining Neu5Acα2-3Galβ1-4GlcNAc glycotope, while ECA prefers to bind the Galβ1-4GlcNAc glycotope present after desialylation. **(B)** Virus binding curves were generated similarly as described in the legend to Fig 2 using 3’S(LN)_2_-PAA. **(C)** Binding of MAL I after incubation of the sensors with the indicated viruses. MAL I binding in the absence of NA activity (no NA) or after treatment of the sensors with Arthrobacter ureafaciensl NA (AUNA) is also shown. **(D)** Similar as for (C), but now using ECA. Low binding levels of MAL I and high binding levels of ECA correspond with high levels of desialylation.

While SeV displayed clear binding and release with a peak height of more than 2.5 nm for 3’S(LN)_2_-PAA, the peak height for NDV was much lower (Fig 2). This might be explained by reduced interaction of NDV with the receptor-coated surface compared to SeV, or alternatively, NDV might be released very efficiently due to high sialidase activity (Fig 1C). To study this in more detail, we analyzed the modification of the receptor-coated surface by sialidase activity of the different viruses using a lectin binding assay (Fig 3A). We performed a similar assay as shown in Fig 2, using sensors coated to saturation with 3’S(LN)_2_-PAA, resulting in similar binding curves for the three viruses analyzed (Fig 3B) as shown in Fig 2. Subsequently, any remaining virions were removed from the receptor-coated surface using three short low pH washes (referred to as regeneration) [6] and binding of *Maackia amurensis* lectin I (MAL I; specific for Neu5Acα2-3Galβ1-4GlcNAc containing glycans) was analyzed. A subsequent regeneration step to remove MAL I was followed by binding of *Erythrina crista*-galli lectin (ECA), which specifically recognizes glycans containing terminal Galβ1-4GlcNAc. Of note, regeneration of the sensors does not affect the binding of biotinylated receptors to the streptavidin sensor. Low MAL I and high ECA levels indicate high levels of desialylation resulting from sialidase activity. Full desialylation was observed when the glycans were incubated with a high amount of bacterial sialidase as evidenced by the lack of MAL I binding (Fig 3C). Incubation of the sensors with NDV or hPIV3 resulted in most and least desialylation, respectively, with SeV displaying an intermediate phenotype (Fig 3C and 3D). Apparently, the low binding levels observed for NDV result from high sialidase activity, in agreement with the results of the MUNANA assay (Fig 1B) and not from lack of association of NDV with the receptor-coated sensors.

### Effect of sialidase inhibitor on NDV, hPIV3 and SeV binding

**Fig 4:**
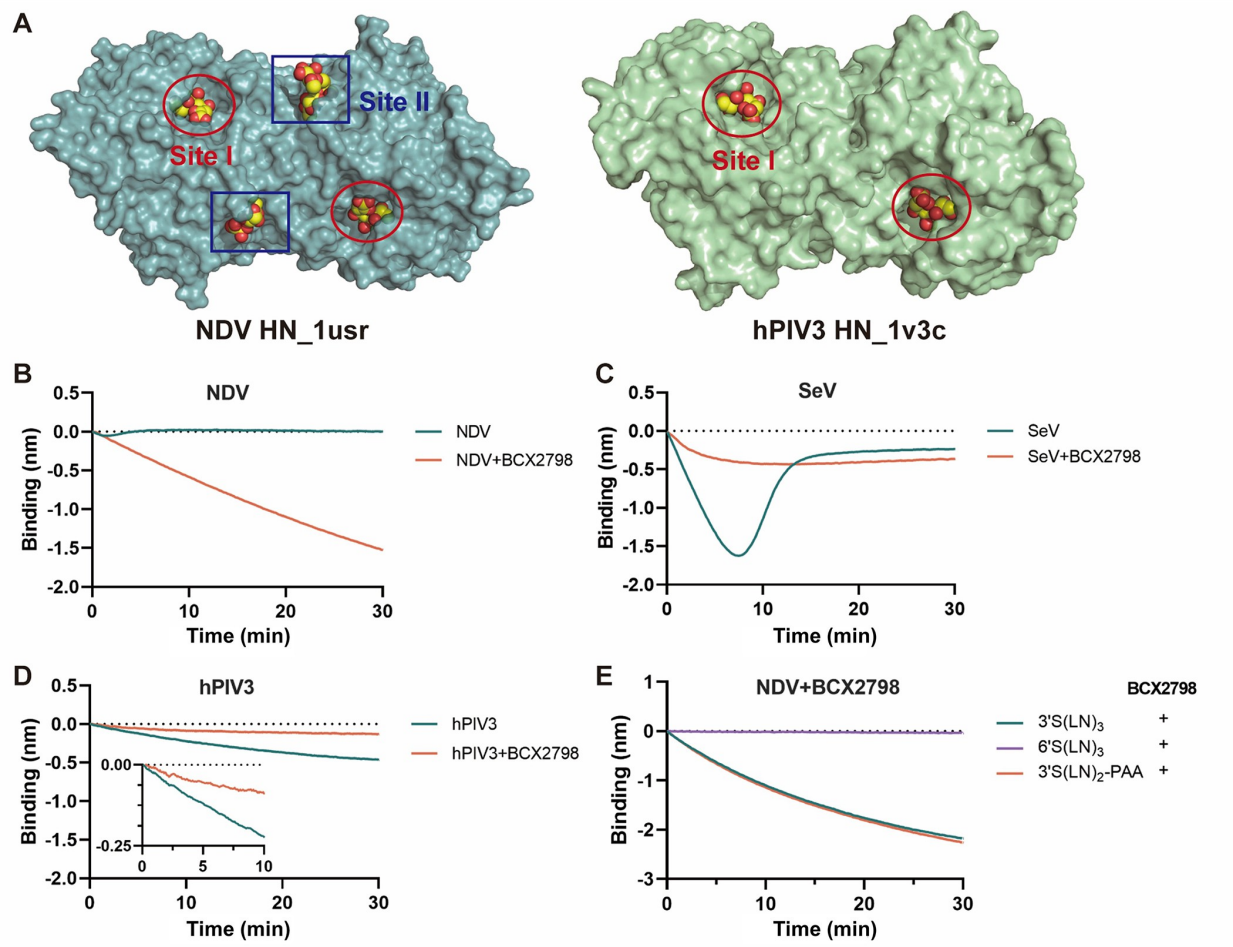
Interaction of NDV, hPIV3, and Sendai virus with 3’S(LN)_2_-PAA in the presence of BCX2798. **(A)** Structure of NDV (PDB 1usr) and hPIV3 (PDB 1v3c) HN dimer is shown in a surface representation. Catalytic site (Site I) and the 2SBS (Site II) occupied with Sia are indicated. Virus binding curves were generated similarly as described in the legend to Fig 2 using 3’S(LN)_2_-PAA for NDV(**B**), hPIV3(**C**), and SeV(**D**) in the absence or presence of 1mM BCX2798. **(E)** Binding of NDV to 3’S(LN)_3_, 6’S(LN)_3_ and 3’S(LN)_2_-PAA in the presence of BCX2798.

NDV HN has a 2SBS located at the dimer interphase [21] (Fig 4A). Such a site does not appear to be present in hPIV3 HN [20,38], while it remains unclear whether SeV contains a 2SBS [22]. The presence of a functional 2SBS was previously demonstrated by the addition of catalytic site inhibitors that do not affect receptor binding via the 2SBS. Here we used the paramyxovirus inhibitor, BCX2798 (4-azido-5-isobutyrylamino-2,3-didehydro-2,3,4,5-tetradeoxy-d-glycero-d-galacto-2-nonulopy-ranosic acid), which design is based on the NDV HN structure (see SI 1 for its chemical synthesis and characterisation details) [39,40]. BCX2798 inhibits cleavage of MUNANA by NDV (IC_50_ value 16.36μM) and SeV (IC_50_ value 0.43μM (S2 Fig). As shown in Fig 4B, the binding of NDV to 3’S(LN)_2_-PAA was increased by the presence of 1mM BCX2798, while no bending of the binding curve was observed, indicative of binding of NDV via its 2SBS in the absence of sialidase activity. A different effect of the inhibitor was observed for SeV and hPIV3. Association of both hPIV3 and SeV was inhibited by BCX2798 (Fig 4C and 4D). A low level binding signal could still be observed for SeV in the presence of BCX2798. Analysis of different receptors showed that NDV prefers binding to α2-3-linked Sia via its 2SBS as it was not able to bind the α2-6-linked sialoglycan 6’S(LN)_3_ in the presence of BCX2798 (Fig 4E). In conclusion, the results are in agreement with NDV having a 2SBS in HN, while the results do not indicate the presence of a similarly functional site in hPIV3 and SeV. The 2SBS of NDV displays a similar receptor preference as the NDV catalytic site, at least for the receptors studied.

### Glycan length and density affects virus-receptor interaction

**Fig 5:**
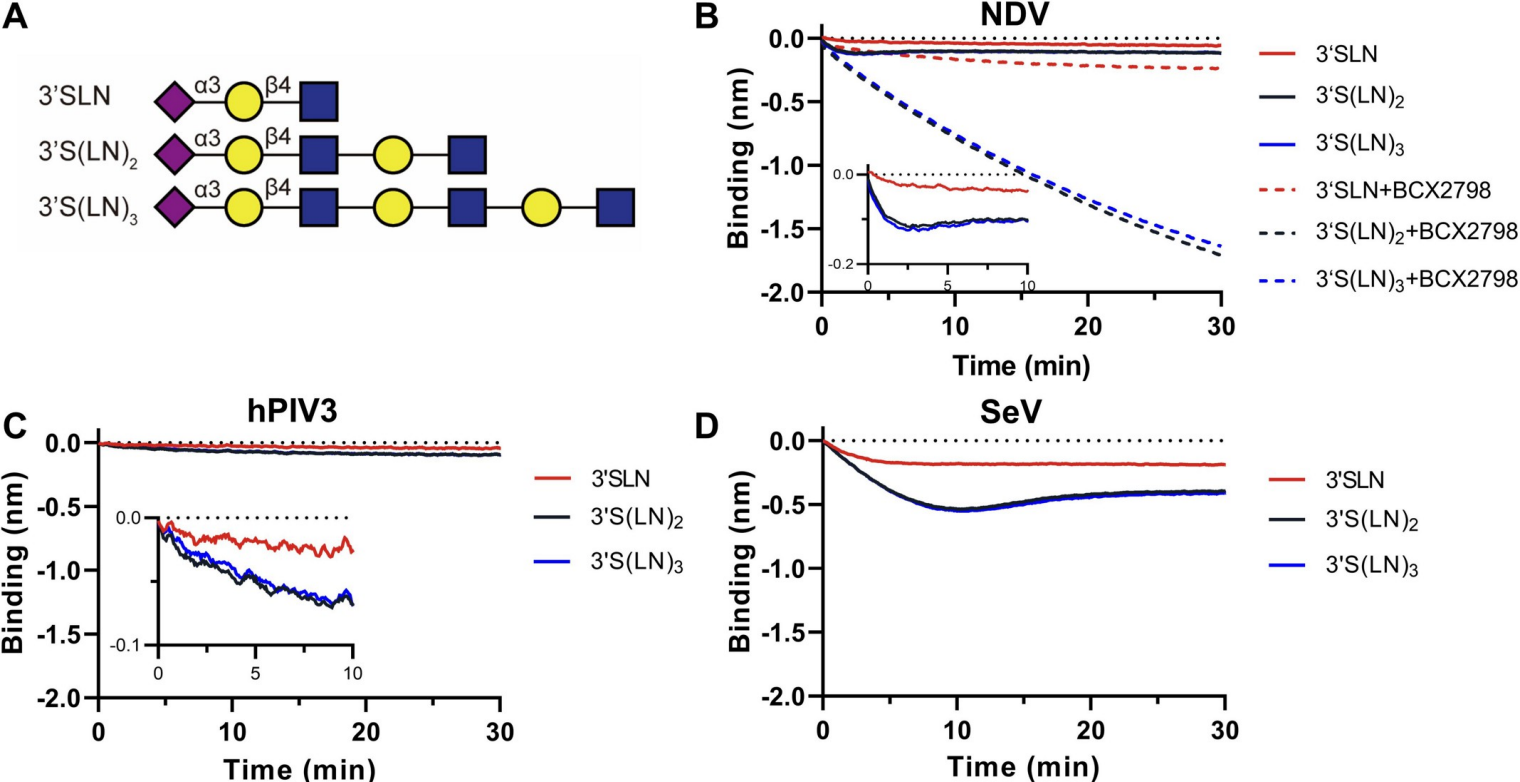
NDV, hPIV3, and SeV interaction with 3’SLN, 3’S(LN)_2_, or 3’S(LN)_3_. (**A)** Schematic representation of biotinylated 3’SLN, 3’S(LN)_2_,and 3’S(LN)_3_ glycan structure. (**B)** Analysis of NDV’s ability to bind to 3’SLN, 3’S(LN)_2_, or 3’S(LN)_3_ in the presence or absence of BCX2798. Analysis of the ability of hPIV3 (**C**) and SeV (**D**) binding ability to 3’SLN, 3’S(LN)_2_, or 3’S(LN)_3_. Virus binding curves were generated similarly as described in the legend to Fig 2.

Glycan length has been shown to be an important determinant of IAV-receptor interactions [8,41–43]. Here we tested whether this also holds true for paramyxovirus-receptor interactions. Sialosides containing one, two or three Gal-GlcNAc (LN) repeats (Fig 5A) were analyzed in BLI analysis. All three viruses displayed similar binding kinetics to 3’S(LN)_2_ and 3’S(LN)_3_, while binding was reduced for 3’SLN (Fig 5B, 5C and 5D). In the presence of BCX2798, a similar preference of binding to sialoglycans containing two or three LN repeats was observed for NDV as in the absence of the inhibitor.

**Fig 6:**
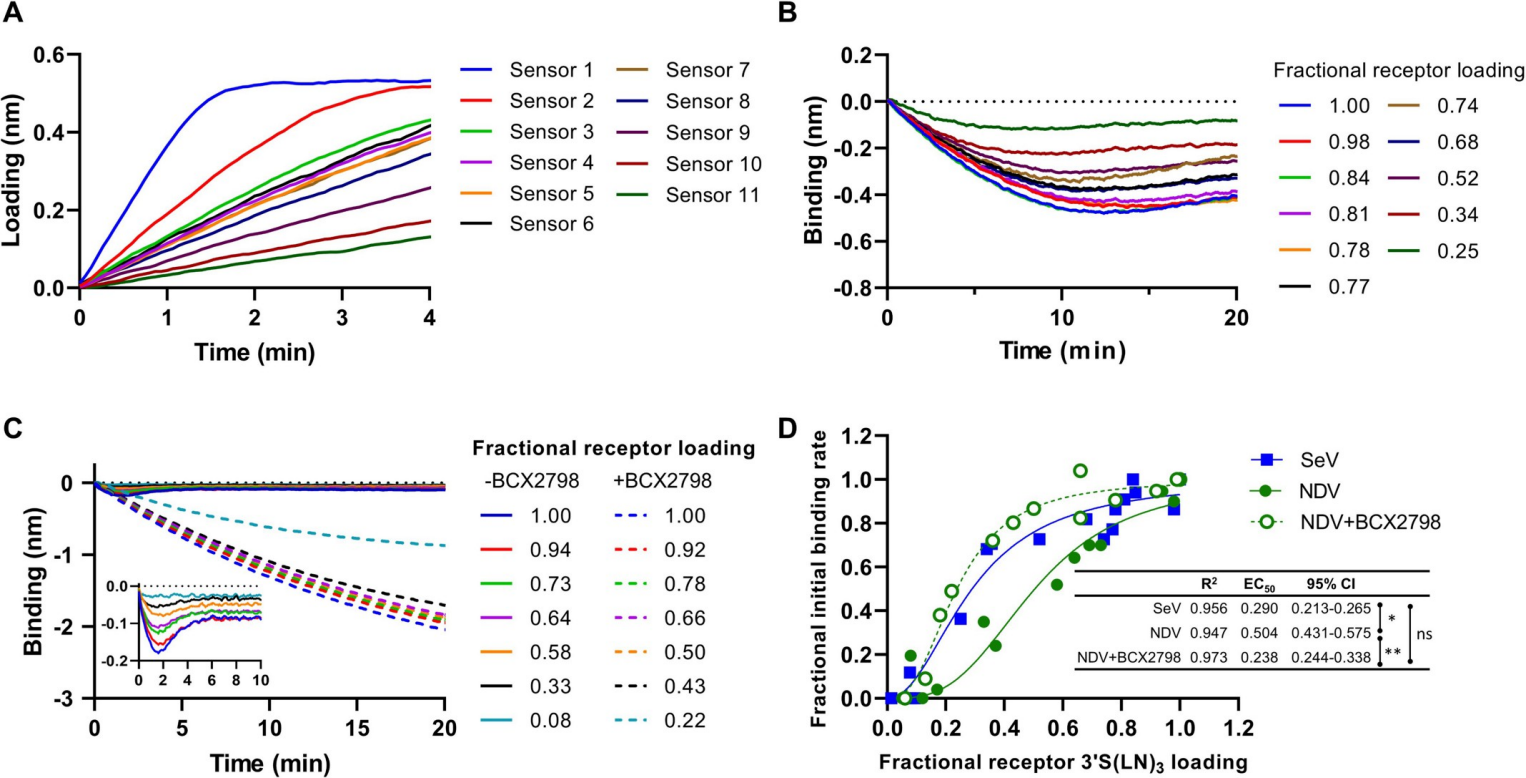
Characterization of NDV and SeV binding kinetics through BLI. (A) Sensors were loaded with different densities of 3’S(LN)_3_ receptor by incubation with different concentrations of receptor. Fractional receptor loading levels were obtained by normalizing receptor binding signals to the signal of sensors containing saturating receptor levels (e.g. sensor 1). Subsequently, sensors were incubated with SeV (B) or with NDV in the presence or absence of BCX2798 (C). The initial binding rate, corresponding to the steepness of the tangent at the beginning of the binding curves was determined form graphs B and C and normalized to the maximal, saturating initial binding rate for each condition, resulting in a fractional initial binding rate. (D) The fractional initial binding rates for SeV, NDV and NDV+BCX2798 were plotted against the fractional receptor density for two independent experiments. Using Graphpad prism software, EC_50_ values and 95% confidence intervals (CI) were determined by nonlinear regression analysis (sigmoidal 4PL, R^2^ values are indicated). Significant differences between EC_50_ values (one-way ANOVA with Tukey’s multiple comparisons test; *, p<0.05; **, p<0.01) are indicated.

Next, we analyzed the effect of different 3’S(LN)_3_ receptor densities (Fig 6A) on the binding kinetics of NDV and SeV (Fig 6B and 6C). The initial binding rates (steepness of the binding curves immediately after starting the virus-receptor binding phase of the experiment) [6] were determined for SeV in the absence of BCX2798 and for NDV in the absence or presence of BCX2798 and normalized to the maximum initial binding rates observed under these conditions. As the low binding levels of hPIV3 did not allow for accurate determination of the initial binding rate at lower densities, this virus was not taken along in the analysis. NDV displayed a higher relative initial binding rate in the presence of BCX2798 than in its absence at low receptor densities (Fig 6D), resulting in lower receptor density at which the half maximum initial binding rate is observed. The curve generated for SeV was similar to that of NDV in the presence of BCX2798.

### Effect of virus particle number on 3’S(LN)_3_ and 3’S(LN)_2_-PAA binding

**Fig 7:**
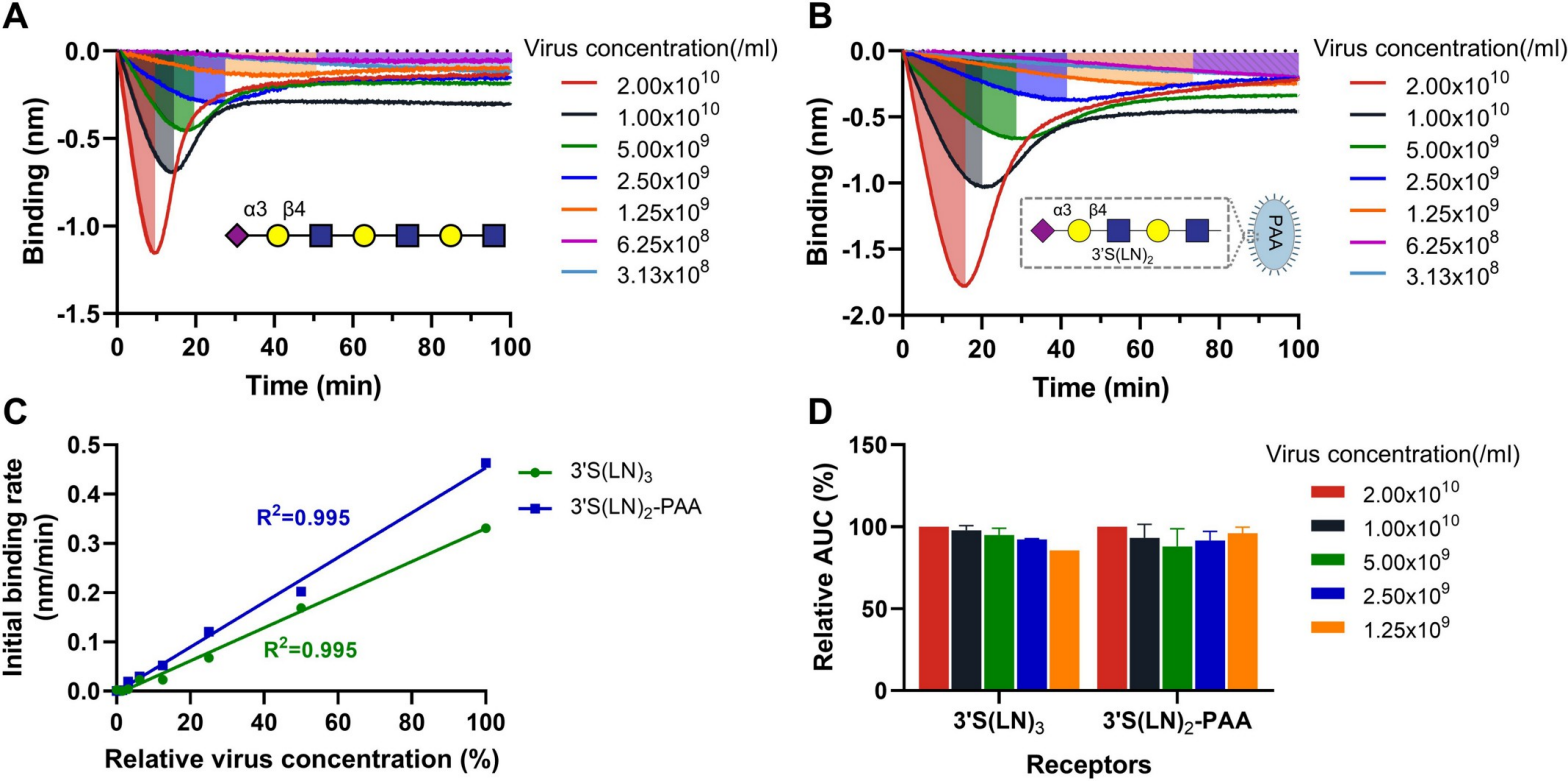
Virion concentration-dependent interaction with receptor-coated surfaces. Streptavidin sensors loaded to saturation with biotinylated 3’S(LN)_3_ (A) or 3’S(LN)_2_-PAA (B) were incubated with SeV at different concentrations for 100 mins. (C) The observed initial binding rates (nm/min) of SeV were plotted against relative virus concentration (100% corresponds to 2.0x10^10^particles/ml) and linear regression analysis was performed. (D) The area under the curve (AUC; from start to peak) was determined at those virus concentrations that displayed peak binding values within this time frame. Means and standard deviations of two independent replicate experiments using different virus stocks are shown.

**Fig 8:**
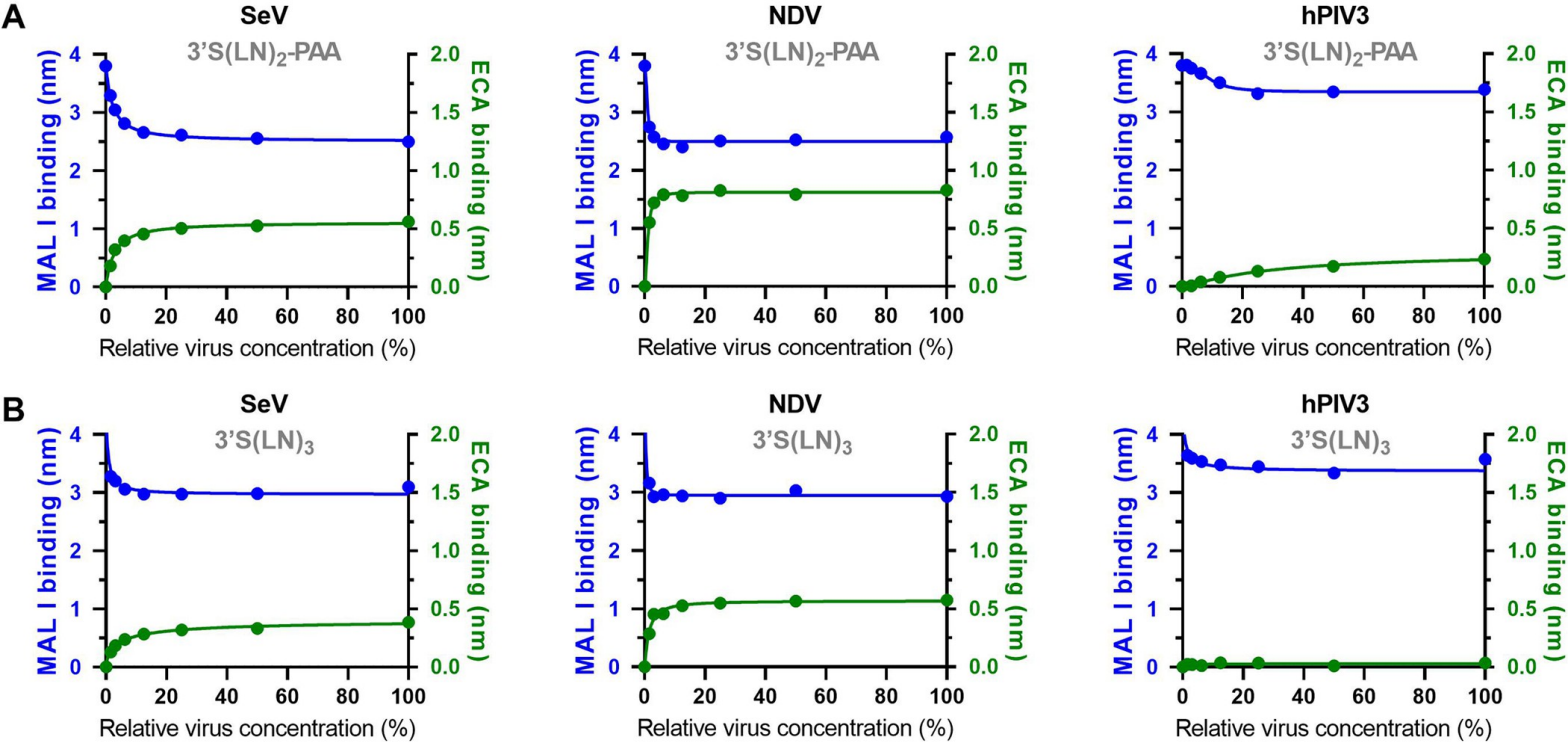
Characterization of receptor-surface modification by viruses using MAL I and ECA lectins. Different concentrations of SeV, NDV or hPIV3 virus particles were allowed to interact with (**A**) 3’S(LN)_2_-PAA- or (**B**) 3’S(LN)_3_- loaded sensors for 30 min (NDV and SeV) or 1h (hPIV3), followed by sensor regeneration in 10mM Tris/Glycine buffer (pH2). Presence of remaining sialoglycans was probed by analyzing binding with MAL I (left Y-axes; blue data) or ECA (right Y axes; green data).The highest virus concentration used (100%) corresponds to 2x10^10^ particles/ml.

To study the effect of virus concentration on the kinetics of virus-receptor interactions, sensors loaded to saturation with either 3’S(LN)_3_ or 3’S(LN)_3_-PAA were incubated with different concentrations of SeV (Fig 7A and 7B). Plotting the observed initial binding rate of SeV to the two receptors against the relative virus concentration, revealed a directly proportional correlation (Fig 7C). While peak heights were higher and reached faster with higher virion concentrations, the area under the curve (AUC; binding signal over time from start to peak) (Fig 7D) was similar at different virion concentrations. Next we analyzed the extent of receptor-coated sensor surface modification by incubation with the different concentrations of SeV by analyzing MAL I and ECA lectin binding as described above. MAL I and ECA binding levels decreased and increased, respectively, with increasing virion concentration (Fig 8A and 8B), until a certain sialoglycan threshold, which was already achieved at approximately 20% of the maximal virion concentration analyzed. The low binding levels of NDV and hPIV3 did not allow an accurate analysis of the initial binding rate at different virus concentrations as shown for SeV in Fig 7. Nevertheless, analysis of the modification of the receptor-coated surface by these viruses using lectin binding showed that also for these viruses the sensor-coated surface was not modified beyond a virus-specific sialoglycan threshold density. As for hPIV3 a clear threshold was not observed after 30 min incubation (S3 Fig), for this virus the incubation time was extended to 1 h. The threshold density was lower for NDV and higher for hPIV3 when compared to SeV, based on the reduction in MAL I binding and the increase in ECA binding.

### Sialidase-dependent and independent virion release

**Fig 9:**
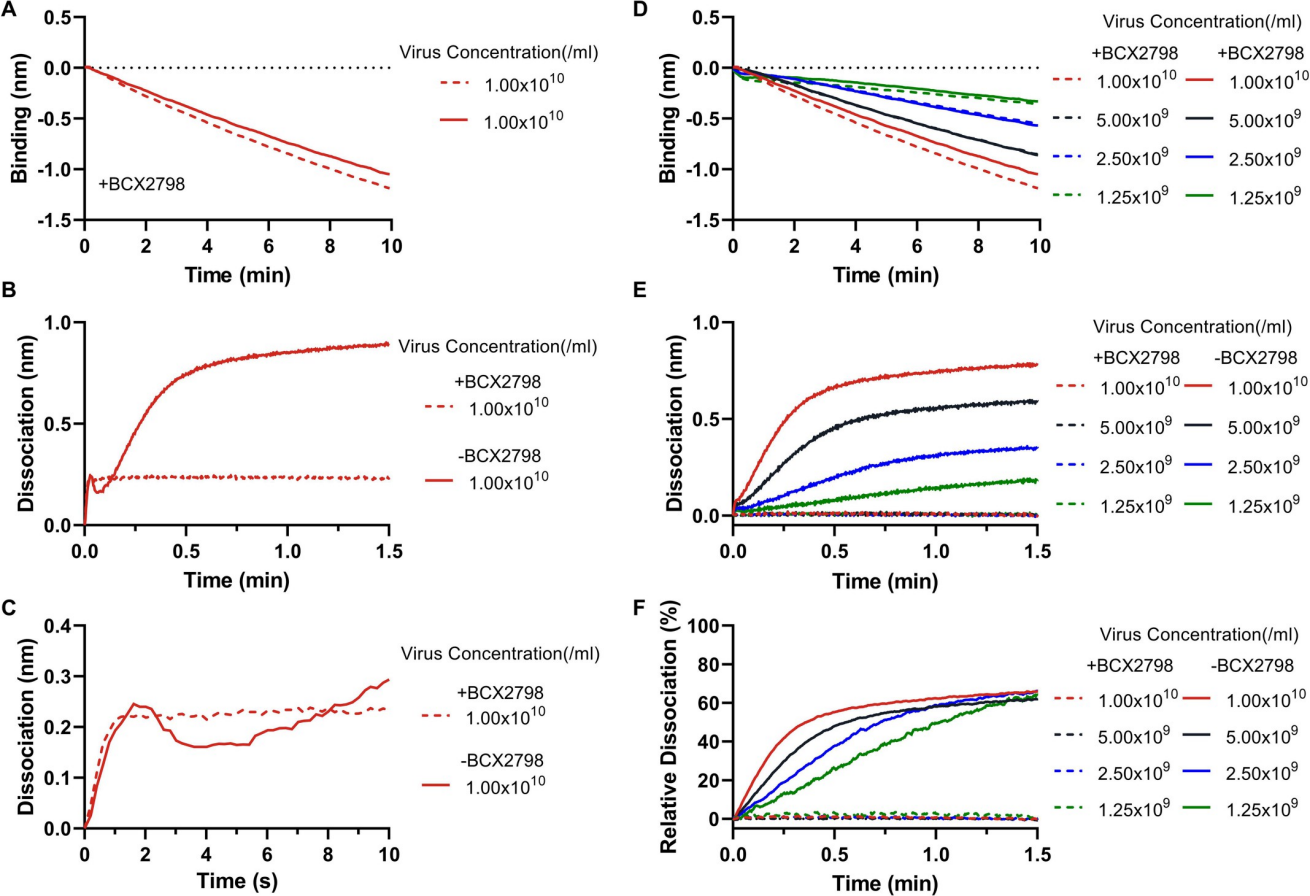
Sialidase-(in)dependent NDV virion release. The streptavidin sensors were loaded to saturation with 3’S(LN)_2_-PAA. **(A)** NDV virions were associated with 3’S(LN)_2_-PAA in presence of BCX2798. **(B and C)** After association to 3’S(LN)_2_-PAA, sensors were dipped into PBS to allow NDV virions to dissociate from the sensor. Panels C is the zoom-in of B in the first 10 seconds. **(D)** Different concentrations of NDV were associated with 3’S(LN)_2_-PAA receptors in the presence of BCX2798. **(E)** Subsequently, sensors were moved to PBS in the absence or presence of BCX2798 to monitor the sialidase-driven virion release. **(F)** Relative sialidase-dependent dissociation curves of NDV virions. Virion dissociation observed in (E) was normalized to the virion binding level observed in (D).

The presence of a 2SBS in NDV (Fig 3A) allowed us to study virion release in the presence and absence of sialidase activity. To this end, NDV was bound to the 3’S(LN)_2_-PAA-coated sensors in the presence of BCX2798 (Fig 9A). Next, sensors were moved to wells containing PBS (with or without BCX2798) and virus dissociation was monitored. In the continued presence of BCX2798 some virion dissociation was observed, which within seconds reached its maximum (Fig 9B and 9C). In the absence of BCX2798, this rapid apparently sialidase-independent release was followed by a short-lived and minor increase in the binding signal, similar to what was observed previously for IAV and which was attributed to a change in virion morphology resulting from a decreasing number of virion-receptor interactions [8], followed by fast release of virus particles that was almost complete within 1.5 min. We conclude that the majority of NDV virions remain attached to a receptor-coated surface in the absence of sialidase activity. In the presence of sialidase activity, these particles are efficiently released.

The observation that modification of a receptor-coated surface by virions is limited to a certain desialylation threshold, which is already reached at low virus concentrations, is suggestive of virion motility on a receptor-coated surface. To confirm this apparent virion motility we next studied to what extent sialidase activity-driven release depended on virus particle concentration. If viruses display sialidase-driven motility on a receptor-coated surface, virion release is predicted to be cooperative, i.e. relatively faster when more particles are bound to the sensor. To study this, sensors loaded to saturation with 3’S(LN)_2_-PAA receptor were incubated with different concentrations of NDV in the presence of BCX2798 for 10 min, resulting in different levels of virion association (Fig 9D). Sensors were then moved to PBS in the absence or presence of BCX2798, and sialidase-driven virion release was monitored (Fig 9E). This release was faster when more virus particles were loaded to the sensors, also when the sialidase-dependent release was normalized for the virus loading level (Fig 9F). From these results we conclude there is indeed cooperativity in the sialidase-driven release of NDV.

### Effect of pH on NDV and SeV receptor association and dissociation

**Fig 10:**
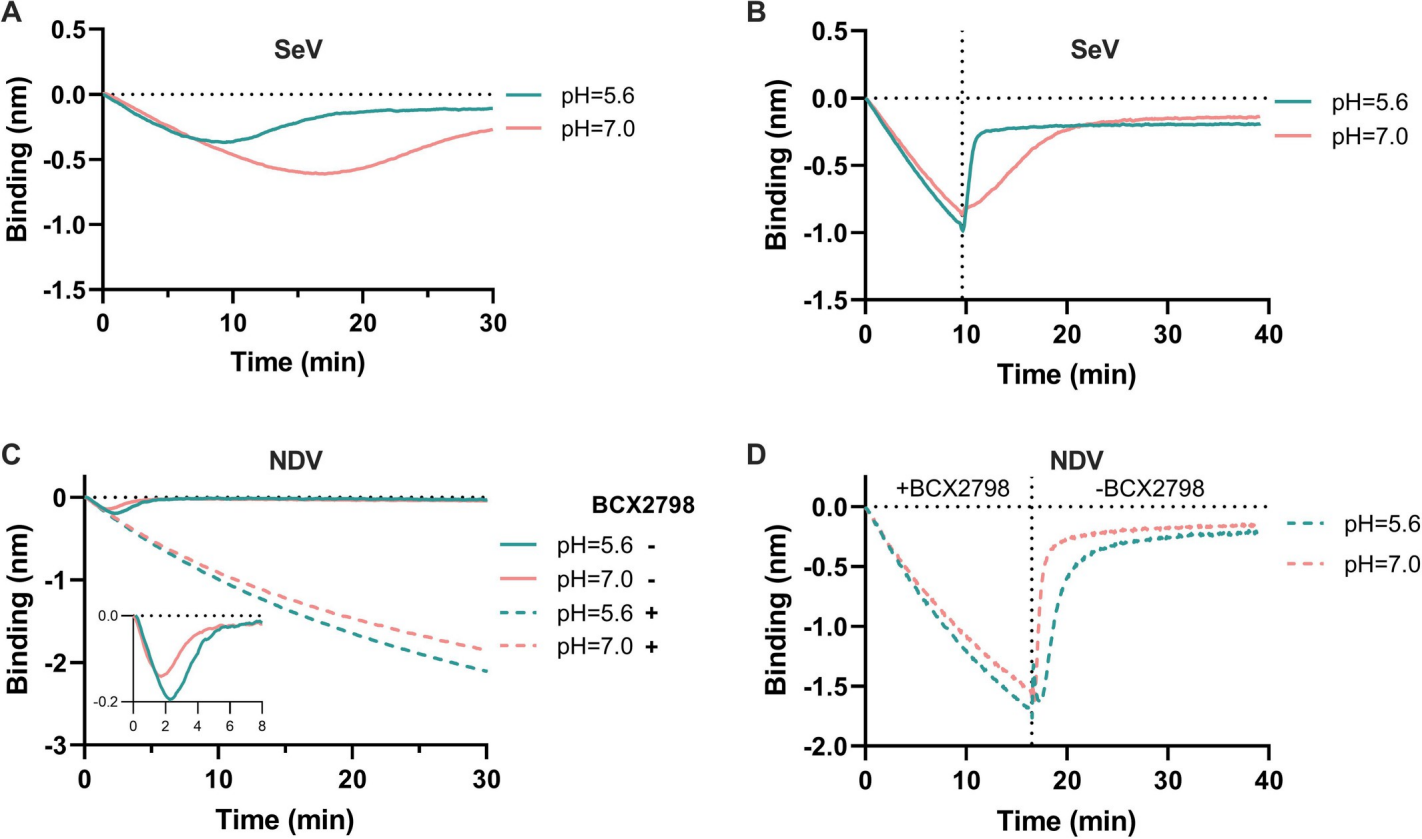
pH effect in SeV and NDV association and dissociation to 3’S(LN)_2_-PAA. **(A)** 3’S(LN)_2_-PAA-loaded sensors were incubated with SeV at pH5.6 or 7.0 for 30 min. (**B)** 3’S(LN)_2_-PAA-loaded sensors were incubated at pH 5.6 or 7.0 with SeV after which sensors were incubated at pH 5.6 or 7.0 in the absence of free virions. **(C)** 3’S(LN)_2_-PAA-loaded sensors were incubated with NDV at pH5.6 or 7.0 in the absence or presence of BCX2798. **(D)** 3’S(LN)_2_-PAA-loaded sensors were incubated with NDV at pH5.6 or 7.0 in the presence of BCX2798. Subsequently, sensors incubated in the absence of BCX2798, allowing sialidase-driven virion dissociation from the sensor.

pH has been reported to be important for sialidase activity of paramyxoviruses in several studies [44–49]. In this study low pH was shown to increase sialidase activity of SeV, although not for NDV (Fig 1B). We therefore analyzed to what extent association and dissociation of virions were affected by pH. SeV displayed pH-dependent interaction to 3’S(LN)_2_-PAA (Fig 10A), which was also observed when using 3’S(LN)_3_ receptors (S4 Fig). While the initial binding rate did not appear to be affected by pH, the virus-binding curve bended earlier at low pH, resulting in a lower AUC. A similar result was observed, when the sensors were moved to wells not containing free virions after 10 min, with virion-release being faster at low pH (Fig 10B and S4 Fig). Also NDV displayed a pH-dependency in its interaction with a receptor-coated surface. While the initial binding rate was not appreciably affected by pH in the presence of BCX (Fig 10C), the AUC was reduced at the higher pH. In agreement herewith, and in contrast to SeV, sialidase-dependent virion release was slower at low pH (Fig 10D). Similar results were obtained for 3’S(LN)_3_-coated sensors (S4 Fig).

## Discussion

**Fig 11:**
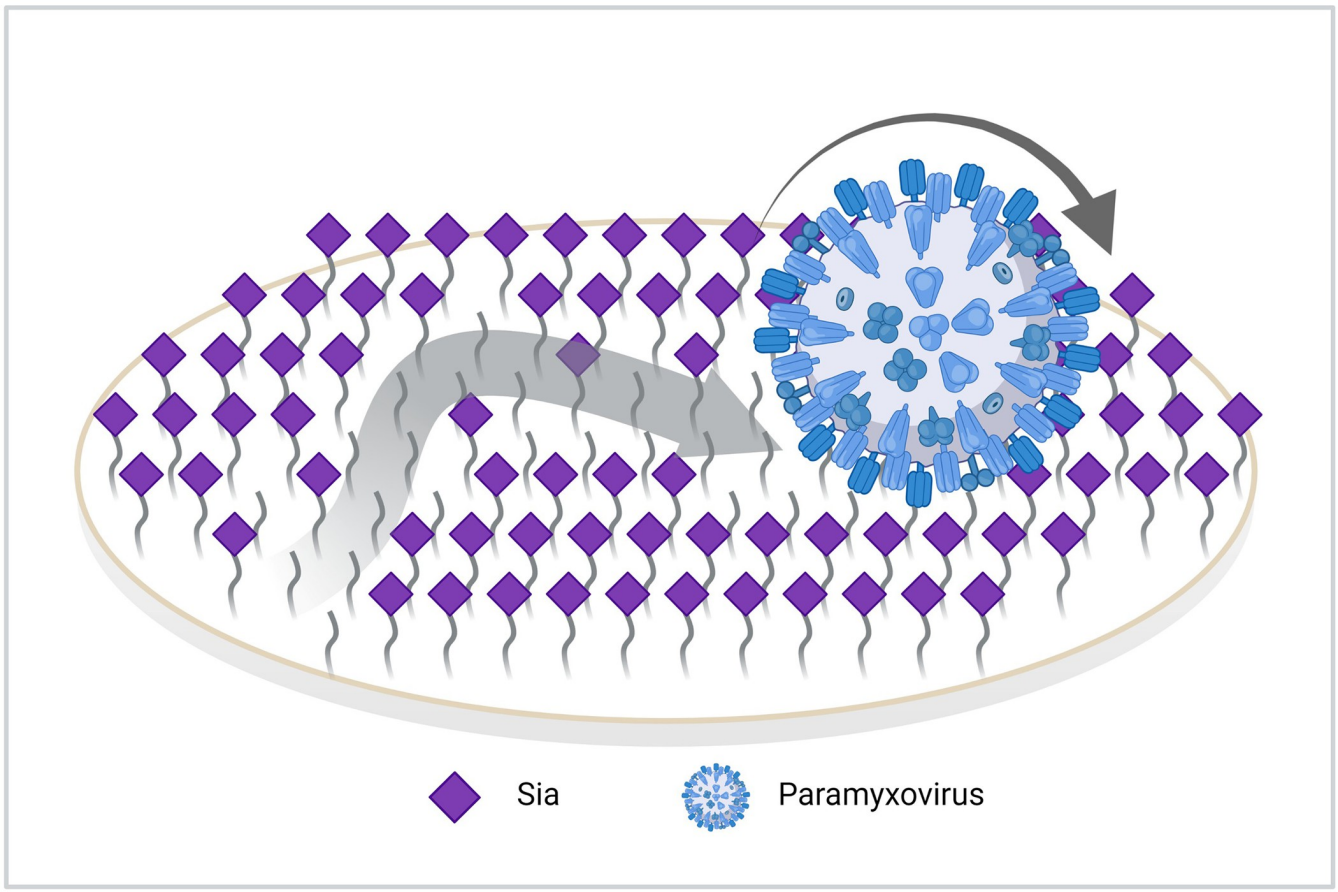
Model for sialidase-driven paramyxovirus particle motility on a receptor-coated surface. Multiple low affinity HN-Sia interactions collectively cause high-avidity binding of paramyxovirions to receptor-coated surfaces. The individual HN-Sia interactions occur via the catalytic site and if present via a 2SBS. These interactions are highly dynamic resulting from low affinity and from cleavage of the sialoglycan receptor. Highly dynamic HN-sialoglycan interactions combined with receptor-destroying activity results in “burnt-bridge”/lawnmower motion [52–54], which is directed by successive cleavage of surface-bound sialoglycans, promoting motion towards unvisited substrate.

Interactions between viruses and sialoglycan receptors are important determinants of virus tropism, replication and pathogenesis [2–5]. These interactions need to be highly dynamic to enable escape from the mucus layer carrying heavily sialylated decoy receptors and to allow virion motility to engage functional entry receptors. The dynamic interactions between virions and receptors are enhanced by these particles carrying receptor-destroying besides receptor-binding activity [28,50]. Sialoglycan-binding paramyxoviruses display these two activities within a single HN protein, and often even via a single active site, which differs from the more-extensively studied influenza A viruses. Here we used BLI to perform kinetic analyses of the interaction of several paramyxovirus particles with receptor-coated surfaces. Among others, these analyses show that different paramyxoviruses, display strikingly different virion-receptor interaction dynamics for the analyzed 3’sialoglycans. These differences correlate with differences in hemagglutination and sialidase activity, and with the presence of a strong 2SBS in NDV, which is lacking in SeV and hPIV3. Viruses displayed cooperativity in sialidase-dependent virion release and modified a receptor-coated surface to a virus-specific sialoglycan threshold level, which was already achieved at low virion concentrations. These results can be explained by paramyxovirus particles displaying sialidase-driven motility on a receptor-coated surface thereby cleaving sialoglycan receptors until a threshold receptor density is reached and virions are released. A similar sialidase-driven motility mode has previously been observed for influenza viruses [6,8,10,11,51]. This virion motility is reminiscent of the motility of nanoparticles referred to as burnt-bridge ratchet or lawnmower motors [52–54] (Fig 11).

The different paramyxoviruses studied here all bound α2-3-linked Sia receptors. Only hPIV3 additionally bound an α2-6-linked receptor, in agreement with results reported by others [55]. These results largely correspond with the notion that sialoglycan-binding paramyxoviruses generally prefer binding to α2-3-linked Sia [34,55–57]. Preferred binding of multiple LN-repeat containing glycans was observed for all viruses tested and is shared with influenza A viruses [8,41–43] and may correspond with increased accessibility of the Sia moiety and the potential of bidentate binding on a single glycoprotein oligomer [43], when extended glycans are immobilized using streptavidin [41]. In addition, structural studies of NDV and hPIV3 HN indicate that the catalytic site contains charged and hydrophobic residues which might contribute to shelter and stabilize interaction with a long glycan chain [19,58–60]. The different viruses differed dramatically in their interaction dynamics when they were probed by BLI using α2-3-linked Sia receptors. Binding of NDV to the receptor-coated sensor was short-lived, in agreement with its high receptor cleavage activity as determined using the sialidase assay (Fig 1B). In contrast, hPIV3 displayed a much slower attachment to and release from the sensors (Fig 2D). As a result, while both viruses displayed limited apparent binding of virions to the sensor surface, they clearly differed in the cleavage of sialoglycans coated to the sensor surface (NDV>hPIV3; Fig 3 and Fig 8). In contrast, SeV displayed a prominent binding signal in BLI, but did not modify the sensor surface to the same extent as NDV, reflecting its lower virion-associated sialidase activity when compared to NDV. The low ability of NDV to bind to low receptor densities compared to SeV in the absence of inhibitor (Fig 6), may also reflect the high sialidase activity of NDV. At low receptor density, NDV may have less opportunity than SeV to establish a multivalent and therefor prolonged binding mode as it may be released faster than SeV from the sensor surface due to the high catalytic activity of its HN protein after the initial binding event. These results fit with the model proposed by Air and coworkers [34], in which HN is a sialidase that can hold its substrate long enough to act as a binding protein and to allow for multivalent virion-receptor interactions, the extent of which differs between the three viruses studied. Some H3N2 IAVs with very weak binding HA display hemagglutinating activity via their NA proteins, probably via a similar mechanism [61].

The interaction of paramyxoviruses with a receptor-coated surface is affected by the presence of a 2SBS in HN [21,22,62]. In the presence of BCX2798, an inhibitor specific for the catalytic site, NDV exhibited pronounced receptor binding (Fig 4) attributed to the presence of a 2SBS located at the HN dimer interphase [21]. The 2SBS (site II) displayed a similar receptor-preference as the catalytic site (site I) (Fig 4). When sensors containing NDV particles associated in the presence of BCX2798 were moved to wells containing the inhibitor but no free virions, the large majority of NDV particles were not released from the sensor (Fig 9) indicating that multivalent binding via the 2SBS is sufficient to immobilize particles. Similarly, IAV particles are also immobilized on a receptor-coated surface in the absence of sialidase activity [8]. BCX2798 inhibited receptor binding of both hPIV3 and SeV (Fig 4), although for the latter some low level binding was maintained. The lack of a 2SBS binding site in hPIV3 HN is consistent with previous studies [20,23]. Low levels of binding of SeV to sialoglycans in the presence of BCX2798 might indicate the presence of an alternative, non-catalytic sialoglycan binding site that is not inhibited by BCX2798 and has a much lower affinity than the 2SBS in NDV HN. This might be a 2SBS in SeV HN, the presence of which has been concluded based on separation of hemagglutination and sialidase activities of SeV HN [22], although there is as of yet no structural evidence for the presence of such a site. It has been proposed that the 2SBS in HN is only activated when site I is occupied by certain ligands, such as zanamivir and BCX2798, and serves to stabilize virus-receptor binding via site I [20]. In addition, the 2SBS of paramyxovirus may also be involved in triggering the F protein [20,21]. For IAV NA, the 2SBS enhances its catalytic activity by bringing multivalent substrates in close proximity of the catalytic site [63]. Whether this holds true for the 2SBS in HN remains to be established.

Our observations indicate that paramyxovirions are motile on a receptor-coated surface in a sialidase-dependent manner, like nanoparticle lawnmowers on a lawn (Fig 11). First, incubation of sensors with reduced particle numbers resulted in lower peak heights at later timepoints, which also took more time to return to baseline levels. The AUC until the peak times were, however, remarkably similar at different SeV concentrations (Fig 7). From our influenza A virus work [8] we know that only viruses bound to the sensor surface (via multivalent interactions) are detected by BLI and contribute to cleavage of sialoglycans, and we assume this to be similar for paramyxoviruses. The independence of the AUC on virion concentration indicates that when fewer virions are associated to the sensor surface, they stay proportionally longer on this surface. These results fit a model in which modification of the sensor surface occurs concomitantly with virion motility, like a lawnmower mowing a lawn. The time needed to modify the sensor surface (to mow the lawn) being inversely proportional to number of virions (lawnmowers) associated to the sensor. Second, the AUC being constant and independent of the virion concentration also suggests that the sensor surface is modified to the same extent for dissociation to exceed association. In agreement herewith, subsequent analysis of receptor cleavage by lectin binding indicated that low (non-saturating [8]) concentrations of virions can modify a receptor surface to the same extent as high concentrations (Fig 8). Virions move on the sensor surface, thereby grazing accessible sialoglycans (Fig 11), until they are released at a virus-specific sialoglycan threshold level and no longer able to modify the sensor surface. hPIV3 with low binding/sialidase activity needed a longer incubation time to reach this threshold, suggestive that the motility of its virions is slower than that of NDV with high sialidase activity. Third, the relative rate of sialidase-mediated self-elution increases with higher NDV virion concentrations, indicative of self-elution being a cooperative process (Fig 9). These results indicate that virions modify a receptor-coated surface that is larger than the contact area they would occupy if they would be stationary, which can only be explained by virion motility, when assuming equally-distributed sialoglycan on the surface. Of note, the inverse correlation between peak height and peak time, resulting in an AUC independent of virus concentration is also indicative of cooperativity of virion self-elution. Sialidase-driven motility was shown to be affected by pH. Low pH did not affect initial binding rates, but stimulated self-elution of SeV virions (Fig 10B) and reduced the AUC (Fig 10A), in agreement with its sialidase enhancing effect (Fig 1) [44,45]. For NDV, sialidase activity was not affected by pH, at least when analyzed with the MUNANA substrate, but self-elution of particles was slower at low pH (Fig 10D) in agreement with a larger AUC (Fig 10C). Apparently, pH may affect virion motility in a virus-specific manner, the relevance of which for infection *in vivo* remains to be established. Motility of paramyxovirus virions was recently proposed for SeV virions based on enhanced release from supported lipid bilayers by the addition of antibodies [64]. However, this would also be expected for stationary particles engaged in multivalent interactions as individual HN-receptor interactions are highly dynamic due to their low affinity. Whether paramyxoviruses are also capable of virion motility in the absence of sialidase activity remains to be established. It will be of interest to confirm and extend our observations on paramyxovirus particle motility using alternative assays such as microscopical analysis of fluorescent particles, similarly as has been done previously for influenza A viruses [8,10].

Virion motility driven by receptor-destroying activity has been reported for influenza A and C viruses using BLI [8] and/or microscopical assays [10,11] and probably also applies to embecoviruses carrying an hemagglutinin-esterase protein. For these viruses the receptor-binding and -cleavage sites are located on different proteins (influenza A virus, embecoviruses) or spatially separated in a single protein (influenza C virus). We now show that virion motility on a receptor-coated surface is also observed for paramyxoviruses even for those that contain a single receptor-interaction site. Regardless, the common mechanism of motility appears to be a burnt-bridge ratchet or lawnmower molecular motor, in which sialoglycans serve as fuel [52–54] (Fig 11). Although individual receptor interactions by the viral attachment glycoproteins are of low affinity, virions are immobilized due to the multivalent binding in the absence of receptor-destroying activity. Combining the low affinity of the individual interactions with an high off rate (monomeric K_off_ being 0.2 s for hPIV) [34] and virion-associated receptor-destroying activity results in dynamic association of particles, local receptor depletion and movement of particles to a higher receptor density. The motility of virions will be affected by changes in receptor-binding affinity and -destroying activity and will presumably be adapted to the host-specific sialoglycome to enable escape from (mucus) decoy receptors, while allowing attachment to and mobility on host cell surfaces to find the functional entry receptors.

## Material and methods

### Cells and viruses

Lewis lung carcinoma-monkey kidney (LLC-MK2) cells were cultured in Dulbecco’s modified Eagle’ s medium (DMEM) (Thermo Fisher Scientific), supplemented with 10% fetal bovine serum (Biowest), 100 IU/ml Penicillin, and 10 μg/ml Streptomycin (Lonza), at 37°C and 5% CO_2_. NDV (Nobilis ND Clone-30) was bought from MSD Animal Health and directly used in the experiments. Human parainfluenza virus 3 (Washington strain) and Sendai virus (Strain 52) were obtained through BEI resources, NIAID, NIH (NR-3233 and NR-3227). hPIV3 and SeV were propagated in LLC-MK2 cells in Opti-MEM (Thermo Fisher Scientific). 1μg/ml TPCK trypsin (Sigma-Aldrich) was additionally added for efficient multiplication of SeV. Viruses were aliquoted and stored at -80°C until use. The virus titers of the different virus preparations were determined by TCID_50_ assay on LLC-MK2 cells using cytopathogenic effects (hPIV3 and SeV) or fluorescence staining of infected cells (NDV; abcam, ab34402) as read out.

### Nanoparticle tracking analysis (NTA)

NanoSight NS300 instrument (Malvern) was used to quantify virus particle numbers as described in our previous publication [7]. Briefly, the virus solution was diluted in PBS to an appropriate concentration for NTA analysis. The NanoSight NS300 generated five 60-second sample video per analysis. Then the videos were analyzed in the Nanoparticle Tracking analysis 3.0 software to get the quantitative information on both virus particle number and size (S5 Fig). All measurements were performed at 19°C. Each sample was analyzed twice and mean values were used. 1x10^10^ virions in the virus preparations as determined by NTA typically corresponded to approximately 1x10^8^ TCID_50_ units of hPIV3, SeV and NDV.

### Hemagglutination and sialidase assays

Human (Sanquin) and chicken red blood cells (RBCs) were repeatedly washed with cold phosphate-buffered saline with Ca^2+^ and Mg^2+^ (PBS^++^, Lonza) until the supernatant was clear and then suspended to 50% in PBS^++^. Virions were quantified through Nano tracker and diluted to 2x10^10^VPs/ml, subsequently 50μl of virus preparation was added into the first row and serially diluted in PBS^++^ in V-shape 96-well plates. Next, 50μl 0.5% RBCs were added and the assay mixture was incubated at 4°C for 2h and the titer read. The sialidase activity of the virus preparations was analyzed using 2’-(4-Methylumbelliferyl)-α-D-N-acetylneuraminic acid sodium salt (4-MUNANA, Sigma-Aldrich) as described previously [65]. The 4-MUNANA is a fluorescent substrate, which yields free 4-methylumbelliferone (4-MU) fluorescent product when cleaved by virus HN, after which the fluorescence intensity can be quantitated. To this end, 50μl (2x10^10^ VPs/ml) of viral solutions was subjected to 2-fold serial dilutions in reaction buffer (50 mM Tris-HCl, 4 mM CaCl_2_, pH 5.5 or 7.0) in a flat-bottom 96-well black plate (Greiner Bio-One), followed by addition of 50μl of 4-MUNANA substrate (200 μM) to each well. 96-well plates containing virus and 4-MUNANA substrate were incubated at 37°C for 60 min. The reaction was terminated by adding 190 μl stop solution (0.1 M glycine, 25% ethanol, pH 10.7). The fluorescence intensity was recorded with an excitation wavelength of 365 nm and an emission wavelength of 450 nm.

### Biolayer interferometry (BLI)

Virus solutions used for BLI (Octet RED348 [Fortebio]) analysis were first purified using Capto Core 700 beads (GE Healthcare Life Sciences) according to the manufacturer’s instructions and then quantified by NTA. Phosphate-buffered saline with Ca^2+^ and Mg^2+^ (PBS^++^, Lonza) was used as buffer for all BLI experiment, the concentration of Ca^2+^ and Mg^2+^ is 133 mg/L and 100 mg/L respectively. When indicated, pH was adjusted by addition of concentrated HCl, with minimal change in volume. Biotinylated synthetic glycans were kindly provided by Geert-Jan Boons (Department Chemical Biology and Drug Discovery, Utrecht University) (3’SLN, 3’S(LN)_2_, 3’S(LN)_3_, and 6’S(LN)_3_) [8,41] or obtained from the Consortium for Functional Glycomics (CFG; 3’S(LN)_2_-PAA; PA362; molecular weight of 30 KDa with 20% sugar and 5% biotin). Standard streptavidin sensors (SA, Pall-ForteBio) were used for BLI “dip and read” analyses, which were performed similarly as described previously [6]. Briefly, the SA sensors were loaded with indicated synthetic glycans and then incubated in PBS^++^ until the baseline was stable. Next, receptor-loaded sensors were incubated in PBS^++^ containing virions for the indicated time to achieve a binding curve. When indicated, BCX2798 (4-azido-5-isobutyrylamino-2,3-didehydro-2,3,4,5-tetradeoxy-D-glycero-D-galacto-2-nonulopy-ranosic acid, synthesized in house, see S1 File) was added. To remove bound virions or lectins, the sensors were regenerated by three 5 s washes in 10 mM Tris/Glycine buffer (pH 2.0), which preserves the binding of biotinylated receptors [8]. The regeneration step was only performed to analyze MAL I and ECA binding. All experiments were performed 2–4 times at 30°C with at least 2 independently generated virus stocks. Virus binding levels were consistently below the BLI maximum binding level (approximately -10 nm) that we observed for influenza A virus previously [8]. Representative experiments are shown. All correlation analyses were performed by using GraphPad Prism 9.0.0.

## Supporting information

S1 File Synthesis of BCX2798. Description of the synthesis of the inhibitor BCX2798 and its characterization by NMR.
(DOCX)

S1 Fig Negligible Virion binding to bacterial-sialidase treated 3’S(LN)_2_-PAA. Virion binding to 3’S(LN)_2_-PAA treated with Arthrobacter ureafaciens NA (AUNA) was analyzed. The insert in C displays a zoom of the first 10 min.
(TIF)

S2 Fig The effect of BCX2798 on NDV or SeV sialidase activity. Sialidase activity was measured in triplicate using the sialidase activity assay described in Material and Methods and the IC_50_ values were determined by non-linear regression analysis using Graphpad Prism software.
(TIF)

S3 Fig Characterization of receptor-surface modification by hPIV3 using MAL I and ECA lectins. The experiment procedure was same as described in Fig 8, but the hPIV3 was allowed to interact with 3’S(LN)_2_-PAA for 30 min.
(TIF)

S4 Fig pH effect in SeV and NDV association and dissociation to 3’S(LN)_3_. **(A)** 3’S(LN)_3_-loaded sensors were incubated with SeV at pH 5.6 or 7.0. (**B)** 3’S(LN)_3_-loaded sensors were incubated with SeV at pH 5.6 or 7.0 for 10 min, after which the sensors were incubated at different pH in the absence of free virions. **(C)** 3’S(LN)_3_-loaded sensors were incubated with NDV at pH 5.6 or 7.0 in the absence or presence of BCX2798. **(D)** 3’S(LN)_3_-loaded sensors were incubated with NDV at pH 5.6 or 7.0 in the presence of BCX2798, after which sensors were incubated at different pH in the absence of BCX2798 and free virions.
(TIF)

S5 Fig Example of Nanoparticle tracking analysis (NTA). An example of SeV concentration analysis using the NanoSight NS300 instrument is shown. The black line corresponds to the average virion concentration (5 measurements), while the red curve represents the standard deviation. Particle concentration (in 10^10^ particles/ml) and diameter (in nm) are graphed on the Y- and X-axis, respectively.
(TIF)
