# Supplementary material for: Kinetic analysis of paramyxovirus-sialoglycan receptor interactions reveals virion motility
Source: PLoS Pathog. 2023 Mar 27;19(3):e1011273. doi: 10.1371/journal.ppat.1011273 (PMC10079232; doi:10.1371/journal.ppat.1011273)
Supplement: S1 File — Description of the synthesis of the inhibitor BCX2798 and its characterization by NMR. (DOCX) [file ppat.1011273.s001.docx]

**Synthesis of BCX2798.** The inhibitor BCX2798 could be synthesized in four steps from known compound **1** (see Scheme below).^1^ After the full deacetylation of **1** with methanesulfonic acid, the isobutyryl was installed on the C-5 amine by selective acylation with triethylamine in the presence of methanol. The resulting intermediate was fully *O*-acetylated to provide **2** to allow for facile purification. Finally, all esters in **2** were hydrolyzed with aqueous NaOH solution to afford BCX 2798 in a good yield.

**Scheme detailing synthesis of BCX2798. a**: i) MeOH/MsOH, reflux, 24h. ii) Isobutyryl chloride, TEA, DCM/ MeOH, 0 °C→r.t, 2h. iii) Acetic anhydride, pyridine, 16h. 31% over three steps. **b**: 1M NaOH (aq), dioxane/water, 30 min. 91%.

**General information for synthesis BCX2798.** Reagents and dry solvents were purchased from commercial sources and used without further purification. Reactions were monitored using thin layer chromatography (TLC) on aluminum plates precoated with Silica Gel 60 F254 (E. Merck). Developed plates were observed under UV light at 254 nm and then visualized after application of a solution of H_2_SO_4_ in EtOH (5% v/v) and heating. Flash chromatography was performed on Silica Gel 60 (0.040−0.063 mm) using distilled solvents. ^1^H and ^13^C NMR spectra were recorded on a Bruker Avance Neo 600 MHz spectrometer. Chemical shifts (δ) are reported in parts per million (ppm), relative to the residual solvent peak as internal reference [CDCl_3_, 7.26 (s) for ^1^H, 77.0 (t) for ^13^C; D_2_O, 4.79 (s) for ^1^H]. High-resolution mass spectrometry (HRMS) was recorded on an Agilent technologies 6560 ion mobility Q-TOF.

**Methyl 7,8,9-tri-*O*-acetyl-2,6-anhydro-4-azido-3,4,5-trideoxy-5-isobutyramido-d-glycero-d-galacto-non-2-enonate (2)** To a solution of **1** (912.30 mg, 2 mmol) in methanol (70 mL) was added methanesulfonic acid (0.804 mL, 12 mmol). The mixture was refluxed for 24 hours and quenched with triethylamine. After removing the solvent under reduced pressure, the resultant compound was dissolved in a mixture of dichloromethane and methanol (v/v 1/1, 60 mL) with 0.7 mL of triethylamine and cooled to 0 °C. Isobutyryl chloride (0.424 mL, 2 mmol) was added dropwise to the solution at the same temperature. The mixture was gradually warmed to room temperature and stirred for 2 hours. When TLC indicated the reaction was finished, the solvent was concentrated under reduced pressure followed by an acetylation of the crude product with an excess amount of acetic anhydride (10 mL) and pyridine (10 mL) for 16 hours. The mixture was concentrated under reduced pressure and diluted with 100 mL of ethyl acetate, and washed subsequently with water (2×100 mL) and brine (100 mL). The organic phase was dried (MgSO_4_) and concentrated under reduced pressure to afford a residue that was purified with silica gel chromatography (light petroleum ether–ethyl acetate, v/v, 3/2→1/1) to yield **2** as yellowish white foam (300 mg, 31% yield over 3 steps).

^1^H NMR (600 MHz, CDCl_3_) δ 6.04 – 5.91 (m, 2H, N*H*, 3-H), 5.41 (dd, *J* = 5.8, 2.1 Hz, 1H, 7-H), 5.35 – 5.29 (m, 1H, 8-H), 4.71 – 4.57 (m, 3H, 4-H, 6-H, 9-H), 4.21 (dd, *J* = 12.5, 6.3 Hz, 1H, 9-H), 3.81 (s, 3H, OC*H*_3_), 3.76 – 3.64 (m, 1H, 5-H), 2.38 (hept, *J* = 6.9 Hz, 1H, isobutyl C*H*), 2.14 (s, 3H, OAc), 2.06 (d, *J* = 13.6 Hz, 6H, OAc), 1.21 – 1.13 (m, 6H, isobutyl C*H*_3_). ^13^C NMR (151 MHz, CDCl_3_) δ 177.63, 170.59, 170.45, 170.07, 161.60, 145.05, 107.70, 75.14, 70.56, 67.69, 61.89, 57.18, 52.58, 49.35, 35.79, 20.85, 20.77, 20.70, 19.35, 18.98.

**4-Azido-5-isobutyrylamino-2,3-didehydro-2,3,4,5-tetradeoxy-d-glycero-d-galacto-2-nonulopyranosic acid (BCX-2798)** To a solution of **2** (69 mg, 0.143 mmol) in dioxane and water (v/v, 3/1, 7.5 mL) was added 1 mL of 1M aqueous sodium hydroxide solution. The mixture had a pH of 14 and was stirred at room temperature for 30 min, after which it was neutralized with Amberlite® IRC120 H, hydrogen form. The resin was filtered, washed with water and the resulting solution was concentrated to afford the crude product, which was purified with silica gel chromatography (ethyl acetate-methanol-water, v/v/v, 7/2/1) to yield pure **BCX2798** as white solid (45 mg, 91%).

^1^H NMR (600 MHz, D_2_O) δ 5.71 (d, *J* = 2.3 Hz, 1H, 3-H), 4.43 – 4.29 (m, 2H, 4-H, 6-H), 4.22 (t, *J* = 10.1 Hz, 1H, 7-H), 4.01 – 3.86 (m, 2H, 8-H, 9-H), 3.70 – 3.59 (m, 2H, 9-H, 5-H), 2.58 (hept, *J* = 6.9 Hz, 1H, isobutyl, C*H*), 1.16 (dd, *J* = 7.0, 3.6 Hz, 6H, isobutyl C*H*_3_). ^13^C NMR (151 MHz, D_2_O) δ 181.49, 103.47, 75.17, 69.78, 68.09, 66.55, 63.04, 59.30, 47.54, 35.28, 18.63, 18.45. HRMS (ESI^-^): *m/z* calcd. C_13_H_20_N_4_O_7_ for [M-H]^-^ 343,1259, found 343.1258.

^1^H NMR spectrum of **2** (600 MHz, CDCl_3_)
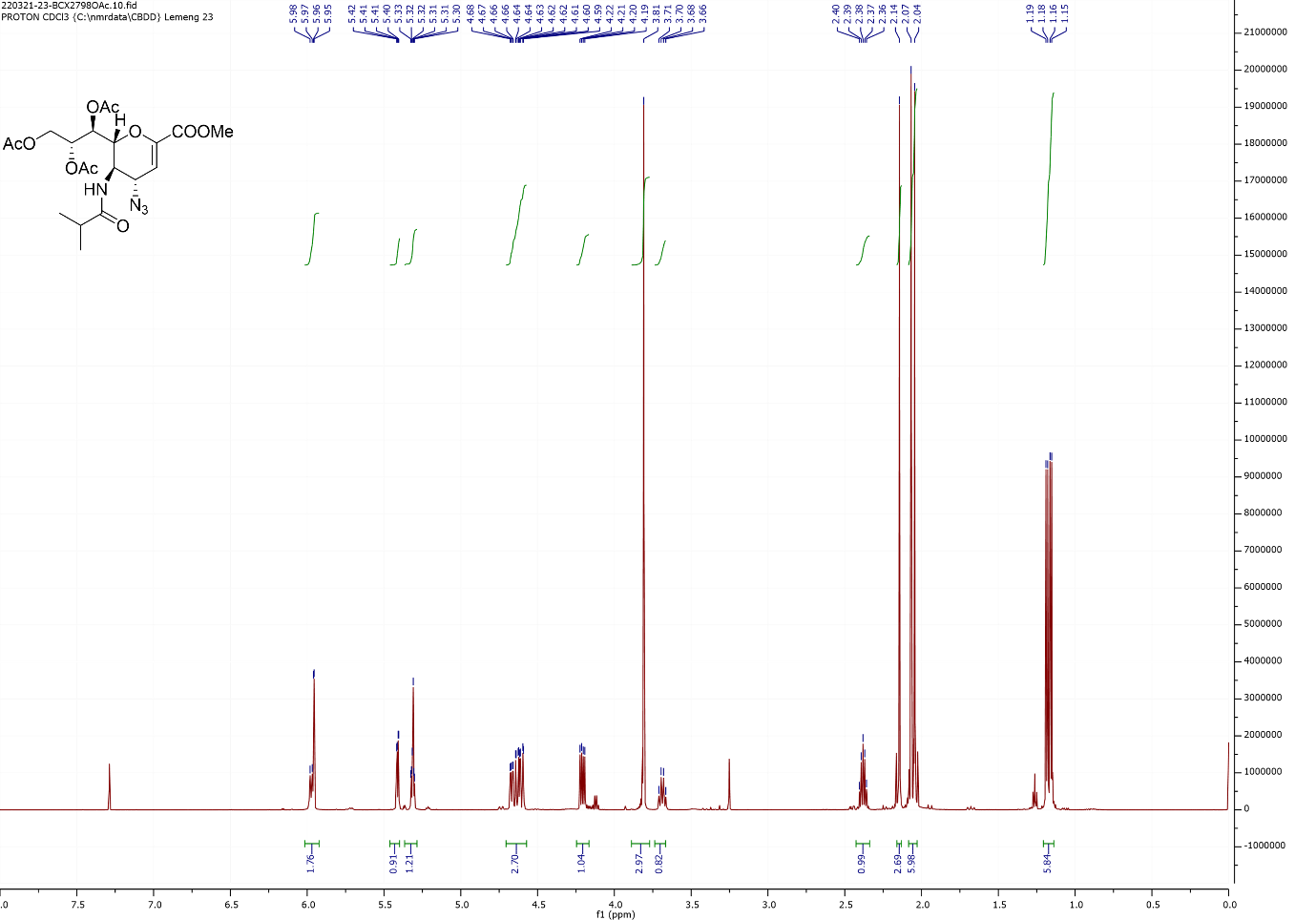


^13^C NMR spectrum of **2** (600 MHz, CDCl3)


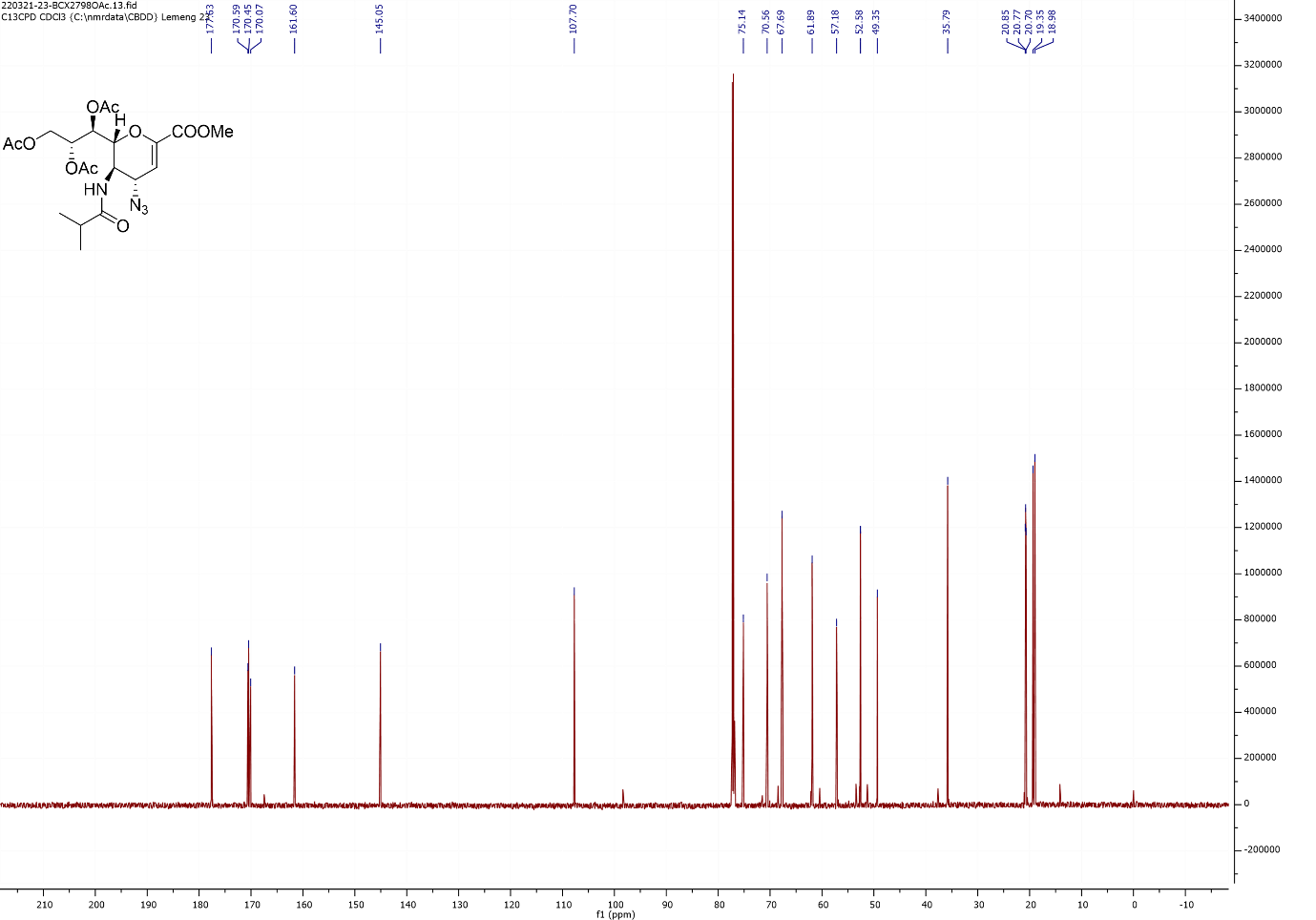


^1^H NMR spectrum of **BCX 2798** (600 MHz, D_2_O)


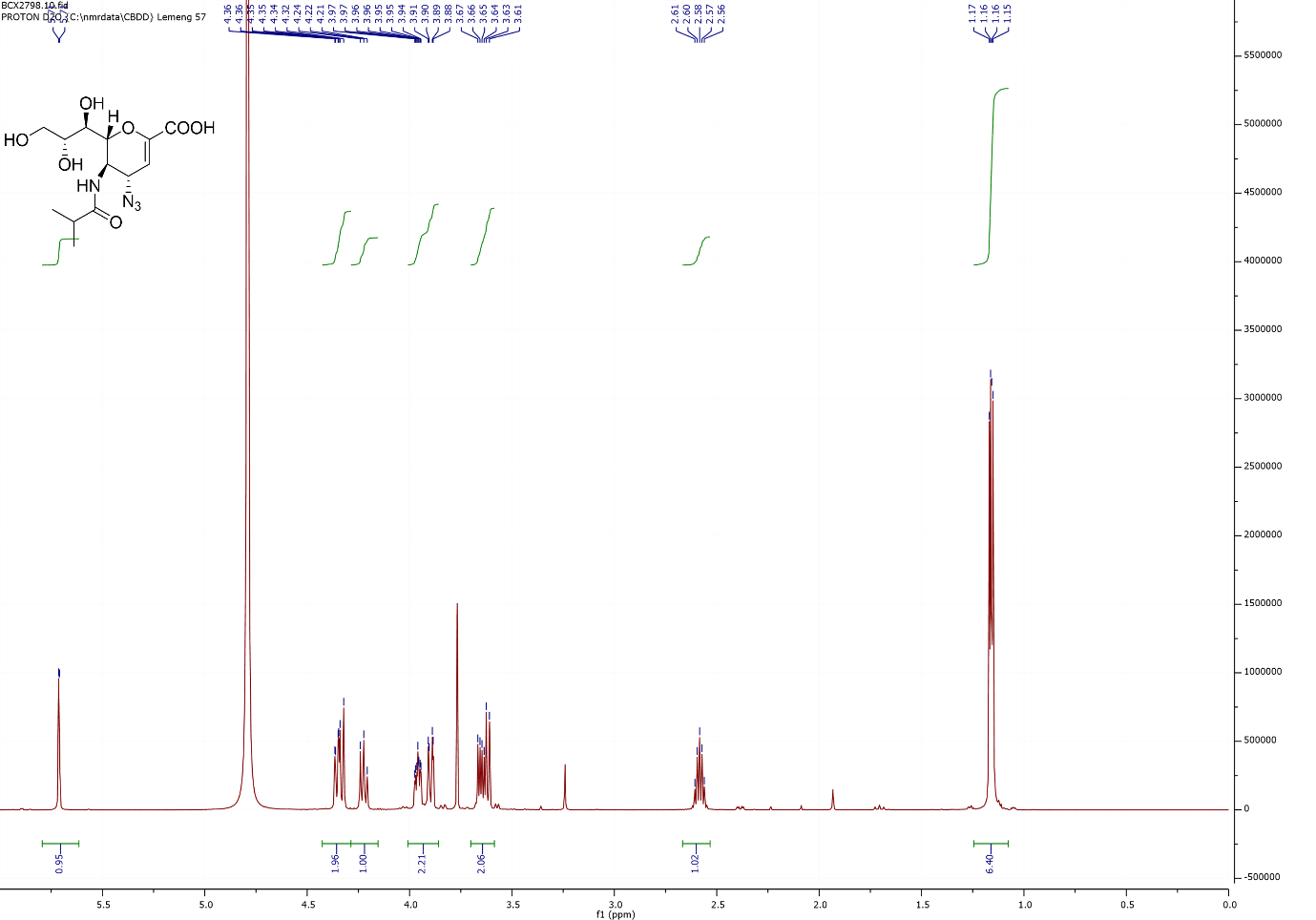


^13^C NMR spectrum of **BCX 2798** (600 MHz, D_2_O)


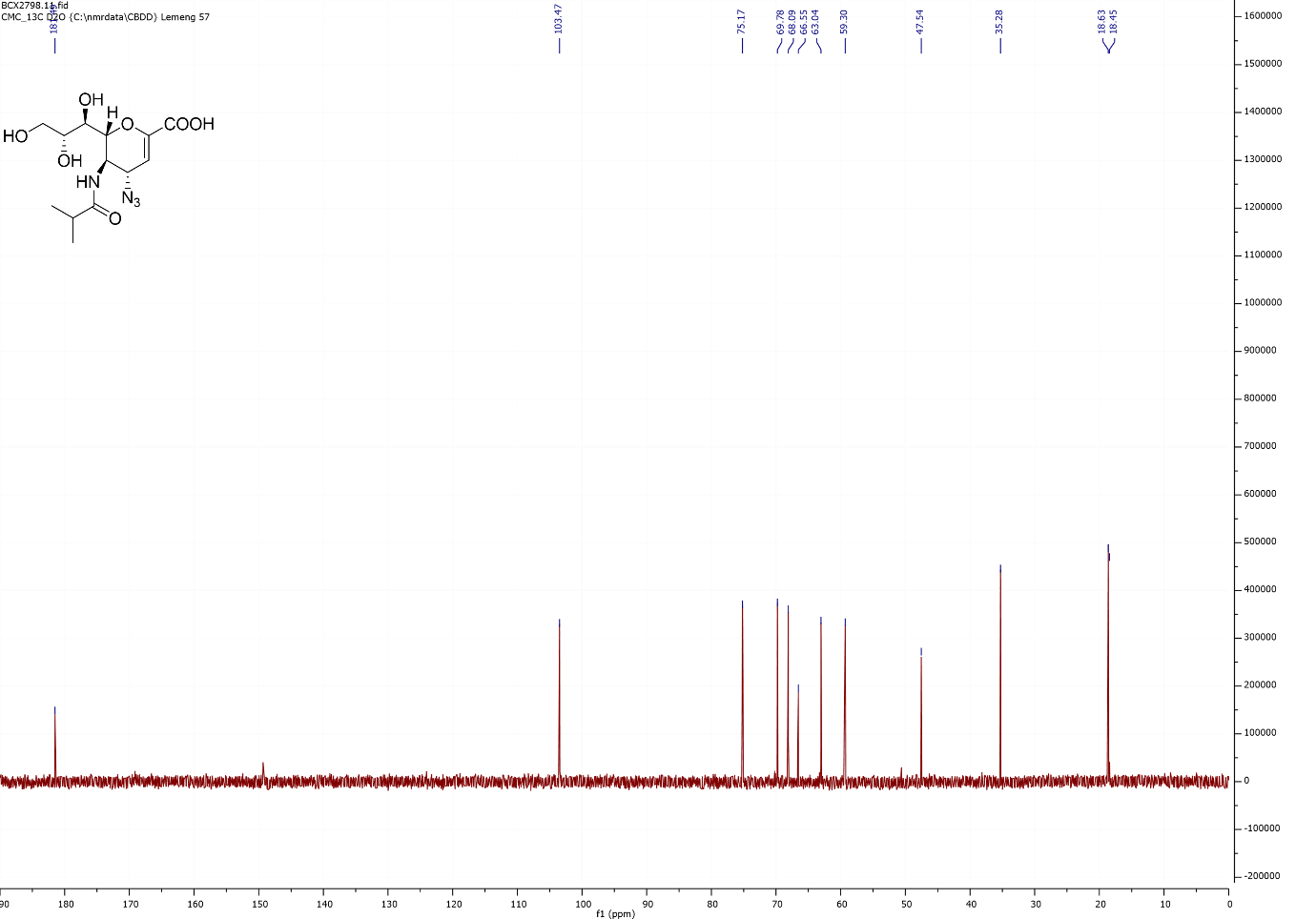


HRMS spectrum of **BCX 2798**


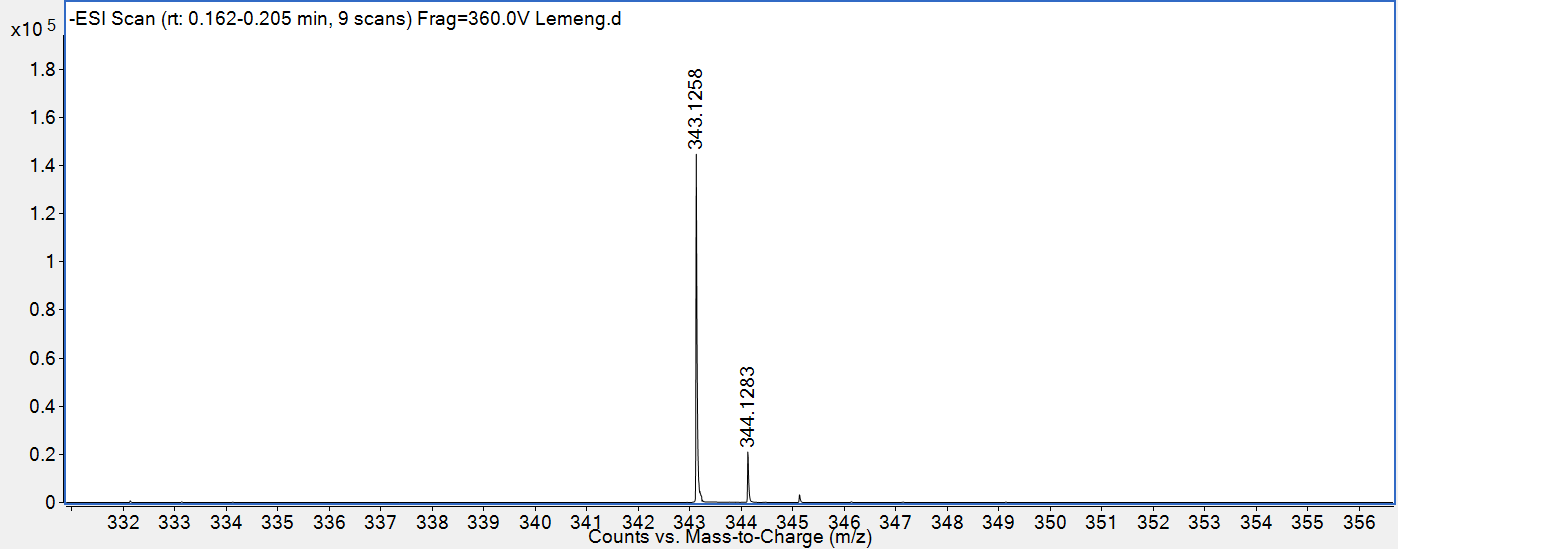


1. Resende, R. *et al.* Mechanism-Based Covalent Neuraminidase Inhibitors with Broad-Spectrum Influenza Antiviral Activity. *Science (80-. ).* **340**, 71–75 (2013).
