# Supplementary figures and images for: Kinetic analysis of paramyxovirus-sialoglycan receptor interactions reveals virion motility
Source: PLoS Pathog. 2023 Mar 27;19(3):e1011273. doi: 10.1371/journal.ppat.1011273 (PMC10079232; doi:10.1371/journal.ppat.1011273)

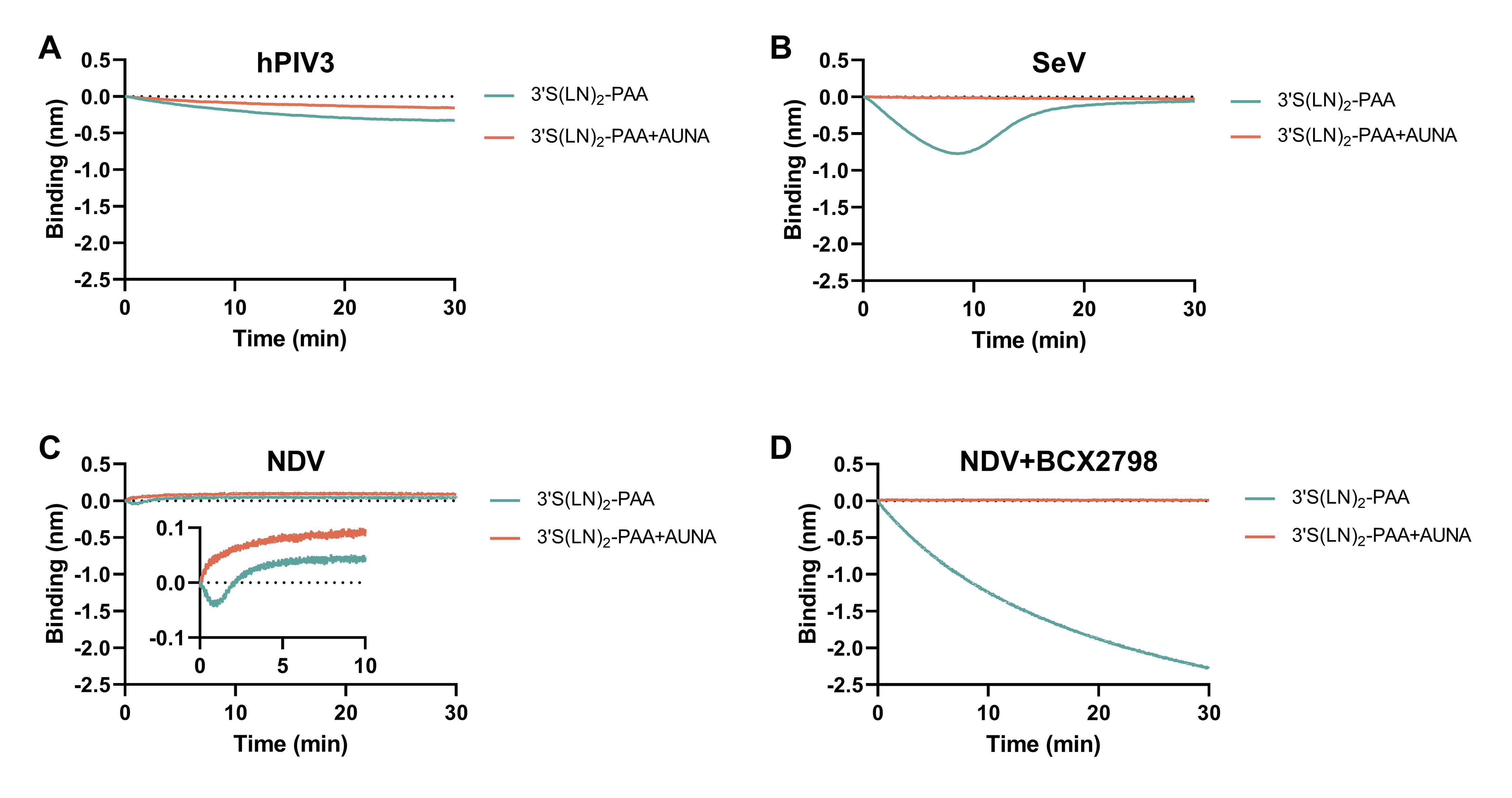

Supplement: S1 Fig — Virion binding to 3’S(LN)2-PAA treated with Arthrobacter ureafaciens NA (AUNA) was analyzed. The insert in C displays a zoom of the first 10 min. (TIF) [file ppat.1011273.s002.tif]

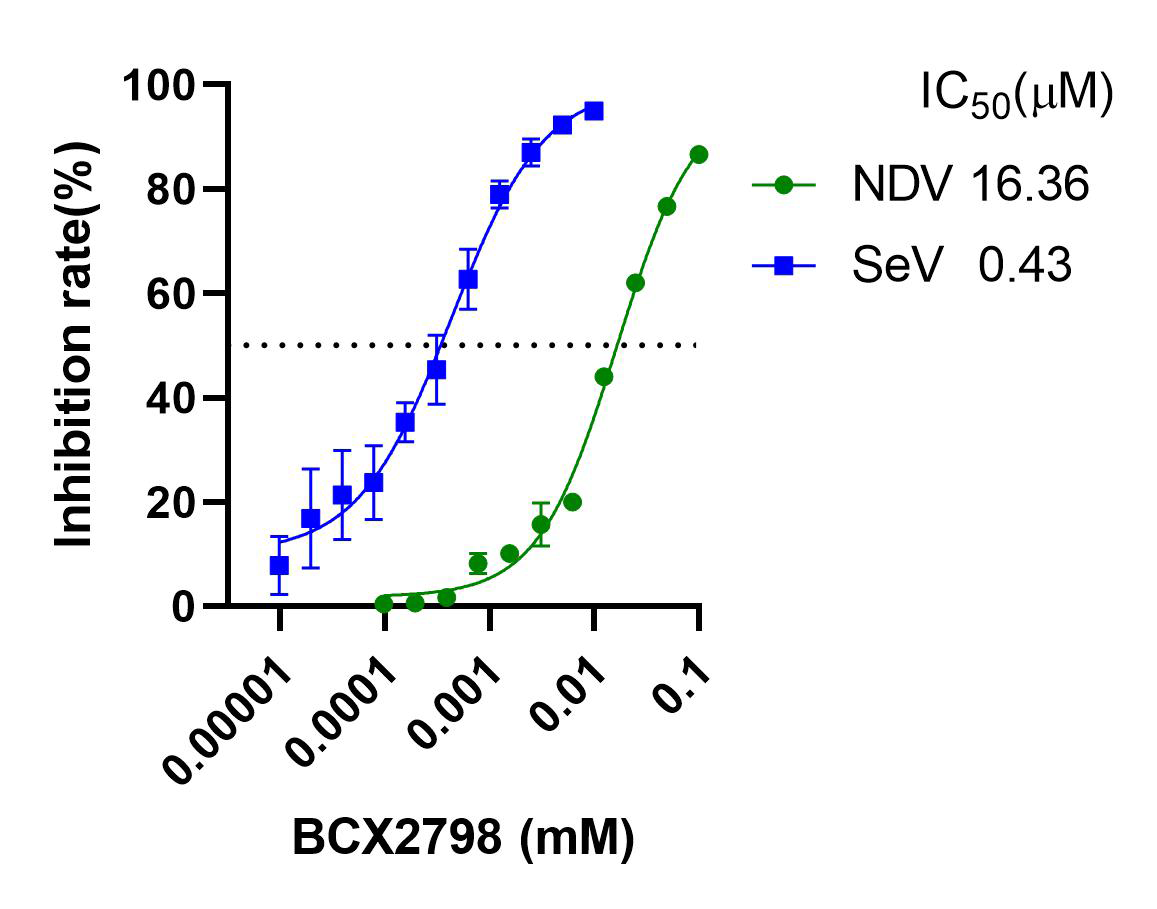

Supplement: S2 Fig — Sialidase activity was measured in triplicate using the sialidase activity assay described in Material and Methods and the IC50 values were determined by non-linear regression analysis using Graphpad Prism software. (TIF) [file ppat.1011273.s003.tif]

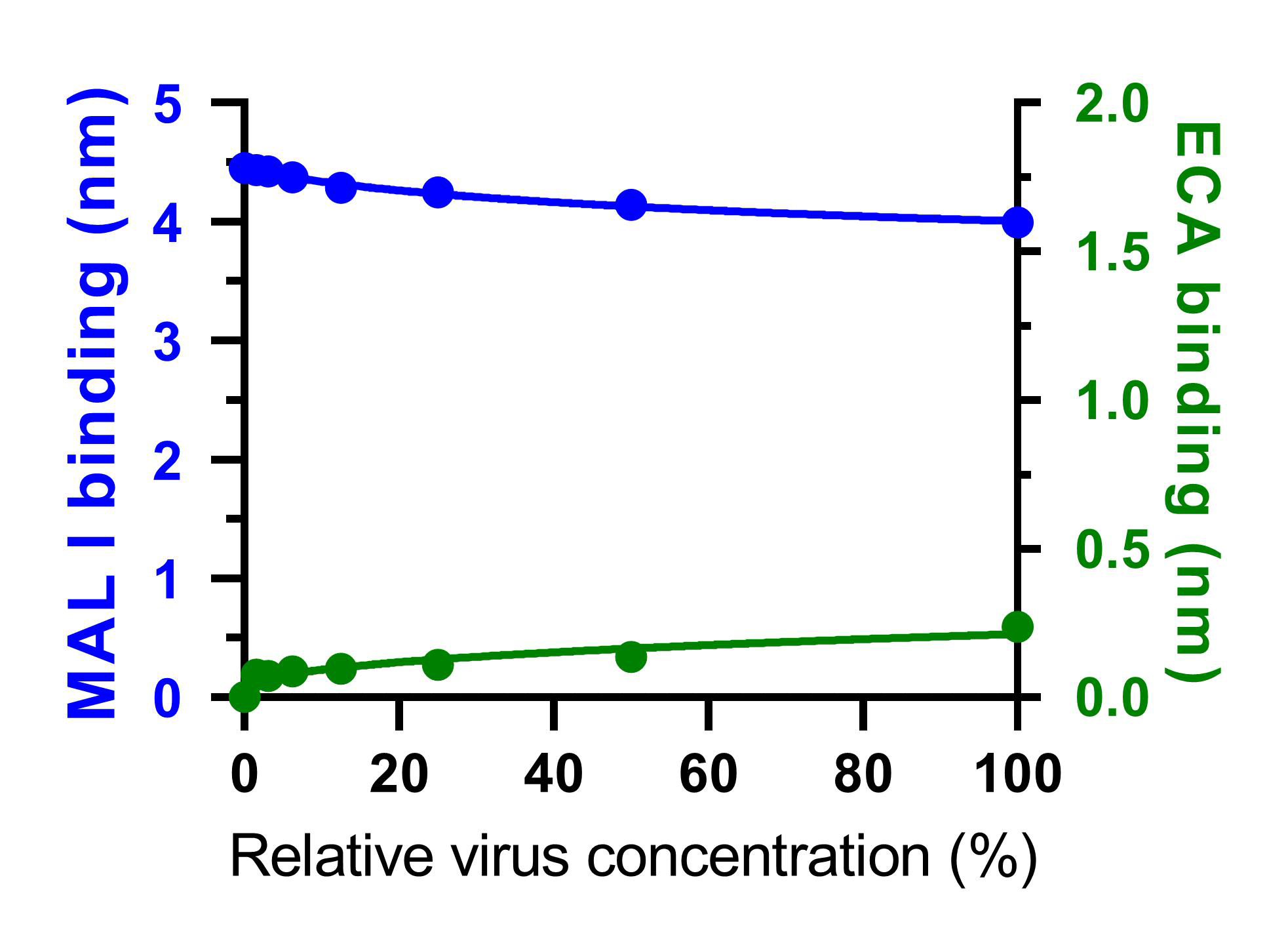

Supplement: S3 Fig — The experiment procedure was same as described in Fig 8, but the hPIV3 was allowed to interact with 3’S(LN)2-PAA for 30 min. (TIF) [file ppat.1011273.s004.tif]

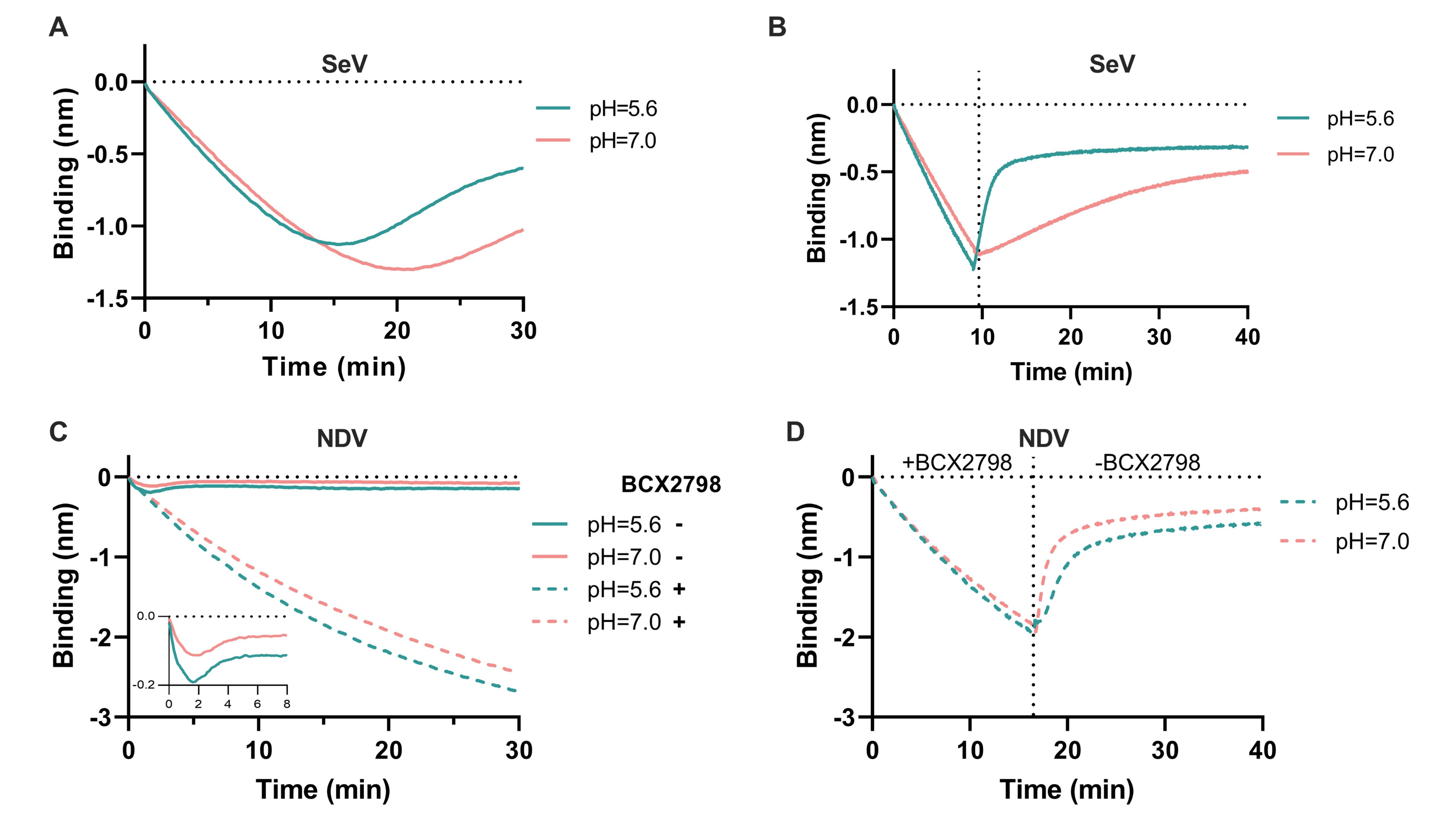

Supplement: S4 Fig — (A) 3’S(LN)3-loaded sensors were incubated with SeV at pH 5.6 or 7.0. (B) 3’S(LN)3-loaded sensors were incubated with SeV at pH 5.6 or 7.0 for 10 min, after which the sensors were incubated at different pH in the absence of free virions. (C) 3’S(LN)3-loaded sensors were incubated with NDV at pH 5.6 or 7.0 in the absence or presence of BCX2798. (D) 3’S(LN)3-loaded sensors were incubated with NDV at pH 5.6 or 7.0 in the presence of BCX2798, after which sensors were incubated at different pH in the absence of BCX2798 and free virions. (TIF) [file ppat.1011273.s005.tif]

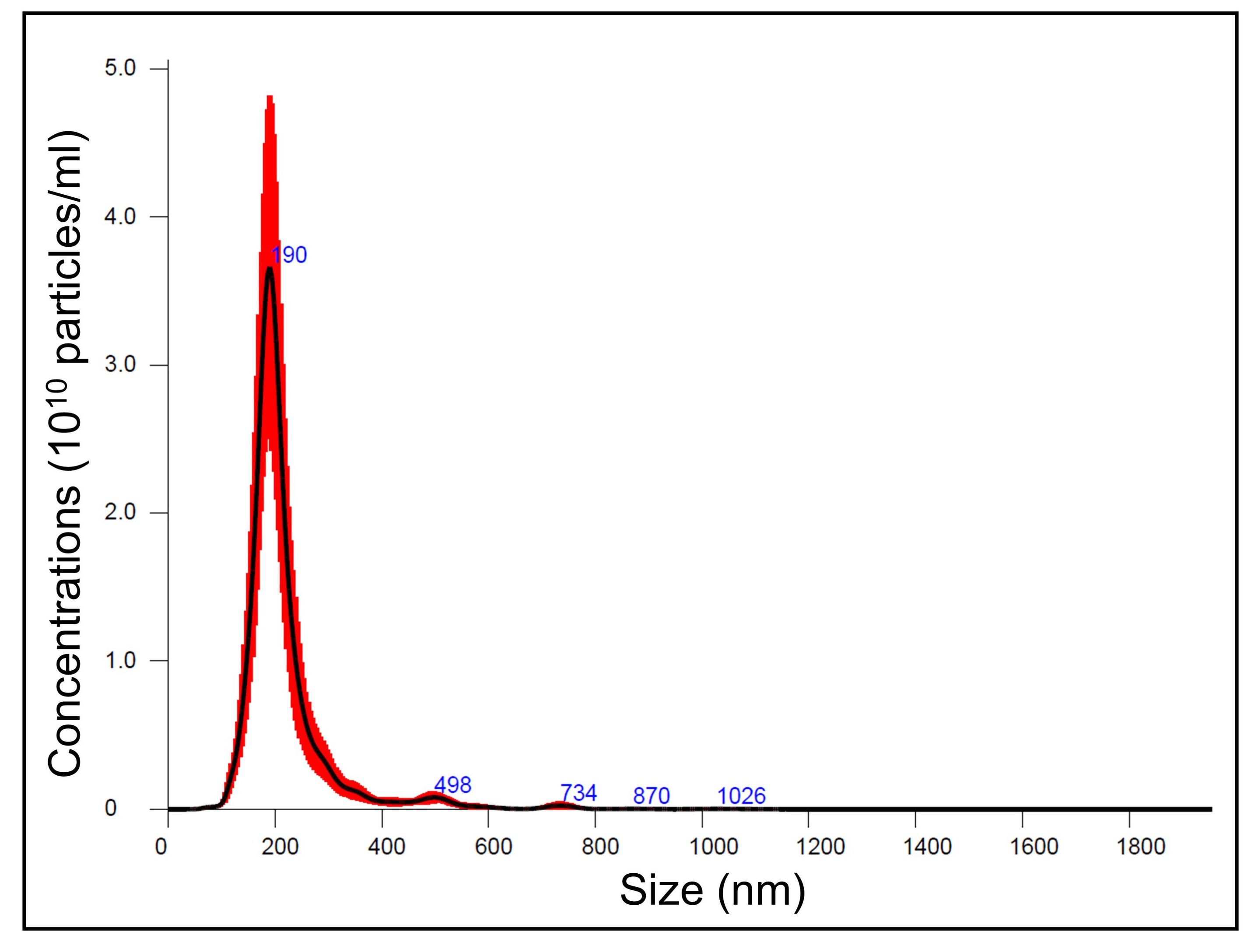

Supplement: S5 Fig — An example of SeV concentration analysis using the NanoSight NS300 instrument is shown. The black line corresponds to the average virion concentration (5 measurements), while the red curve represents the standard deviation. Particle concentration (in 1010 particles/ml) and diameter (in nm) are graphed on the Y- and X-axis, respectively. (TIF) [file ppat.1011273.s006.tif]
